# Metabolic syndrome: molecular mechanisms and therapeutic interventions

**DOI:** 10.1186/s43556-025-00303-5

**Published:** 2025-08-26

**Authors:** Luyao Zheng, Anqi Zeng, Li Liu, Weiwei Tian, Ruirui Wang, Lei Zhang, Hua Hua, Junning Zhao

**Affiliations:** 1 School of Pharmacy, Chengdu University of Traditional Chinese Medicine, Chengdu, 611137 China; 2 Biological Evaluation of TCM Quality of the State Administration of Traditional Chinese Medicine, Translational Chinese Medicine Key Laboratory of Sichuan Province, Sichuan Engineering Technology Research Center of Genuine Regional Drug, Sichuan Provincial Engineering Research Center of Formation Principle and Quality Evaluation of Genuine Medicinal Materials, Sichuan Institute for Translational Chinese Medicine, No. 51, Section 4, Renmin South Road, Wuhou District, Chengdu, 610041 China; 3 Shanghai University of Traditional Chinese Medicine, Shanghai, 201203 China; 4 National Center for Nanoscience and Technology, Beijing, 100190 China

**Keywords:** Metabolic syndrome, Gut microbiota, TCM polyphenols, Short-chain fatty acids, Intestinal barrier inflammation

## Abstract

Metabolic syndrome (MetS, MS) is a group of metabolic disorders characterized by central obesity, insulin resistance, dyslipidemia, and imbalance of glucose homeostasis. Studies have revealed that the molecular mechanism of MetS may be related to adipose dysfunction, insulin resistance, chronic inflammation, oxidative stress, the gut microbiota and epigenetic modifications. At present, the clinical treatment of MetS is limited to lifestyle changes and targeted drugs for a single risk factor, which makes it difficult to achieve the desired effects. Recent studies have shown that the gut microbiota and its metabolites play important roles in various metabolic activities. Polyphenols are the most prevalent chemical components in traditional Chinese medicines (TCMs). TCMs have long been used in the treatment of MetS. TCM polyphenols exhibit significant efficacy in the treatment of MetS by regulating the homeostasis of the gut microbiota, affecting the secretion of its metabolites, and regulating related upstream and downstream pathways such as the AMPK, PPAR, MAPK, PI3K/Akt and NF-κB pathways. This study investigated the molecular mechanisms of MetS and gut microbiota homeostasis in relation to the therapeutic efficacy of TCM polyphenols against MetS. This study further compared TCM polyphenols with existing treatments, thus providing a novel theoretical basis and strategy for MetS treatment and prevention using TCM polyphenols.

## Introduction

With more effective control of communicable diseases worldwide, noncommunicable diseases (NCDs) are becoming a major public health challenge [1]. The incidence of metabolic syndrome (MetS, MS), the most common NCD, is increasing globally, especially in areas where the Western diet is prevalent [2]. Similar trends have been observed in China, the Middle East, India, and Brazil [3]. MetS is a complex group of abnormal metabolic symptoms that are closely associated with cardiovascular disease (CVD), including a state comprising abnormal lipid metabolism, hypertriglyceridemia, lowered high-density lipoprotein (HDL) cholesterol, increased arterial blood pressure, and impaired glucose tolerance [4, 5], which progressively causes hypertension, central obesity, insulin resistance (IR), dyslipidemia, and dysregulation of glucose homeostasis, increasing the risk of individuals developing type 2 diabetes mellitus (T2DM) and CVD [6, 7]. Although the exact pathogenesis of MetS has not yet been fully defined, it is widely recognized to involve a complex interplay of genetic and environmental factors, with hepatic, vascular and immune factors, among others, potentially playing key pivotal roles in the progression of obesity and insulin resistance [8, 9]. In agreement with the research results reported by Vesa et al. [10], we believe that the molecular mechanism of MetS may be linked to adipose dysfunction, insulin resistance, chronic inflammation, oxidative stress, the gut microbiota and epigenetic modifications. At present, the clinical treatment of MetS is limited to lifestyle changes, targeted drug therapy for single risk factors, bariatric surgery and bariatric management. In addition to the above therapeutic interventions, the most common clinical treatment is based on the combination of functional probiotics and other forms of intervention (such as lifestyle changes plus drugs/probiotics and probiotic adjuvant drug therapy). These existing therapeutic methods have several disadvantages and often have difficulty achieving the desired effects.

The human microbiota, especially the abundant gut microbiota, plays a crucial role in maintaining the health of the host [11]. The gut microbiota affects host physiological functions, including the circadian rhythm, nutrient absorption, metabolism, and immune responses, through many complex mechanisms (such as the involvement of metabolites from the gut microbiota in metabolism and the gut‒liver axis) [12–14]. The gut microbiota produces a variety of metabolites, such as trimethylamine-N-oxide (TMAO), short-chain fatty acids (SCFAs), bile acids (BAs), branched-chain amino acids (BCAAs), and tryptophan, which have varying effects on host physiology [15–17]. Traditional Chinese medicines (TCMs) are natural medicines used scientifically based on traditional Chinese medical theory, and the complex ingredients of these medicines have multiple effects on the human body. Polyphenolic compounds in TCMs, which are secondary metabolites of plants, have been demonstrated to exert therapeutic effects on MetS by modulating the gut microbiota composition and related metabolic activities [18, 19]. Based on research into the molecular mechanisms of and treatments for MetS, this review focuses on how TCM polyphenols can influence the progression of MetS by regulating the homeostasis of the gut microbiota and the secretion of its metabolites. In addition to comparing the advantages and disadvantages of traditional treatment methods and TCM polyphenol interventions, this review also provides new perspectives and potential strategies for the treatment of MetS with TCM polyphenols based on clinical cases.

## Molecular mechanisms underlying metabolic syndrome

The concept of MetS first appeared in 1923, when the Swedish scientist Kylin described a syndrome of metabolism-related diseases characterized by hypertension, hyperglycemia and hyperuricemia [20]. The concepts of “insulin resistance (IR) syndrome”, “cardiometabolic syndrome” and “syndrome X” subsequently emerged. In 1988, Reaven described the insulin-stimulated glucose uptake resistance involved in the development of non-insulin-dependent diabetes mellitus (NIDDM), hypertension and coronary artery disease (CAD) as “syndrome x”. Since then, the idea of “metabolic syndrome (MetS)” has been established [21]. Modern pharmacological studies have shown that obesity caused by adipose dysfunction leads to insulin resistance, that the body is in a state of chronic inflammation and oxidative stress over a long period of time, that the homeostasis of the gut microbiota and its metabolites is imbalanced, and that epigenetic modifications mediated by diet, exercise and nutrition promote the generation and development of MetS.

### Gut microbiota disorder and metabolite imbalance

**Fig. 1:**
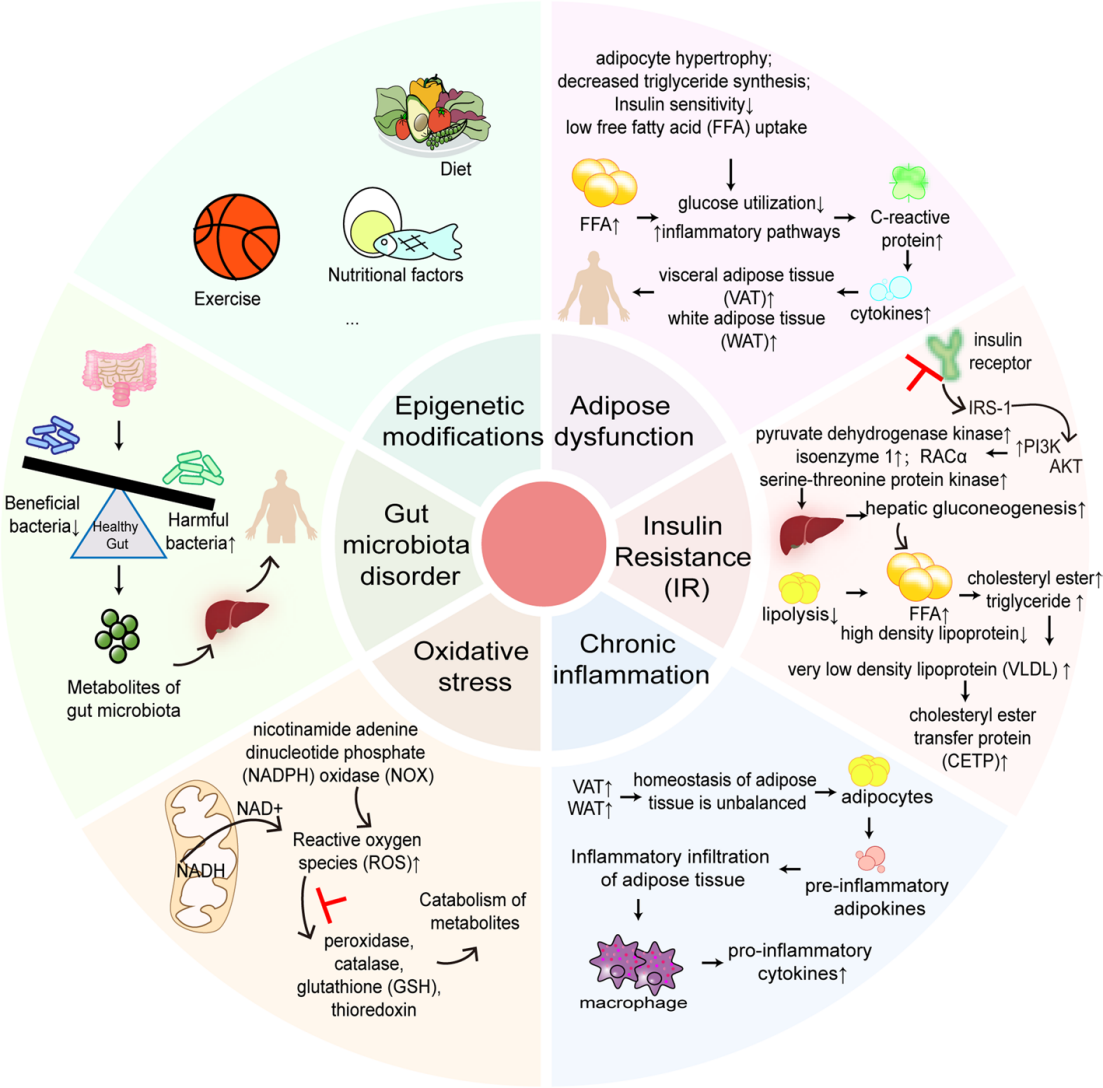
Molecular mechanisms of metabolic syndrome (MetS). Metabolic syndrome may be associated with adipose dysfunction, insulin resistance (IR), chronic inflammation, oxidative stress, the gut microbiota and epigenetic modifications. When adipose dysfunction occurs in the body, abnormal accumulation of body fat leads to an increase in free fatty acids (FFAs) in the peripheral circulation, which affects peripheral glucose intake and activates inflammatory pathways, resulting in obesity. When the body exhibits insulin resistance (IR), the PI3K pathway is activated, which stimulates the production of pyruvate dehydrogenase kinase; reduces the insulin sensitivity of muscle, adipose tissue and the liver; increases liver gluconeogenesis; and disrupts glucose metabolism in the body. In the context of IR, the ability of insulin to inhibit lipolysis is impaired, resulting in an increase in circulating FFAs and the secretion of related lipid metabolism factors. When the body is in a state of chronic inflammation and oxidative stress, the secretion of inflammatory factors and the normal metabolic consumption of reactive oxygen species (ROS) are inhibited. In addition, the homeostasis of the gut microbiota and the secretion of its metabolites also affect the pathogenesis of MetS, and these metabolites can participate in systemic metabolism through the gut‒liver axis. Epigenetic modifications mediated by diet, exercise and nutrition can affect DNA methylation, histone modification and microRNA expression

As the “forgotten organ”, the gut microbiota may play a crucial role in the etiology and development of a variety of diseases. Over the past decade, the gut microbiota has been linked to the onset and development of diseases such as obesity, diabetes, CVD, and cancer. Recent studies have proposed that the gut microbiota is a key regulator affecting disease risk because of its close relationship with metabolism and the immune system [22]. As shown in Fig. 1, the involvement of the gut microbiota in metabolism involves many processes, including the processing of nondigestible dietary polysaccharides into short-chain fatty acids (SCFAs) [23]; the synthesis of vitamins; and the regulation of energy balance, the nervous system, the circadian rhythm and immune function [22, 24]. Researchers have conducted many studies on the effects of the gut microbiota and its metabolites on MetS. Mounting evidence suggests that homeostasis of the gut microbiota may reverse human obesity and metabolic dysfunction. For example, changes in the ratios of *Firmicutes/Bacteroidetes* and *Prevotella/Bacteroides* are readily observed in obese patients, e.g., relative increases in abundance for *Proteobacteria* and *Firmicutes* and decreases in abundance for *Bacteroidetes* and *Akkermansia* [25, 26]. SCFAs, which are metabolites of the gut microbiota, maintain gut barrier stability and function; among them, butyric acid protects pancreatic β-cells via the MAPK and PI3K/Akt pathways [27] and improves insulin sensitivity and secretion by stimulating GLP-1 secretion and reducing inflammation in adipocytes, which in turn alleviates IR [28]. Zhang [29] conducted fecal analysis and reported that, after adding *Edgeworthia gardneri (Wall.) Meisn.* aqueous extract, the relative abundance of *Lachnospiraceae, Rikenellaceae,* and *Dorea* decreased, whereas the relative abundance of *Clostridiales* and *S24-7* increased. Additionally, increased levels of SCFAs in diabetic mice, as observed in feces, inhibited the mucosal infiltration of inflammatory cells in the proximal colon, maintained intestinal barrier stability, protected islet cells, and improved IR. Yan [30] demonstrated that *Anemarrhena asphodeloides* promoted the growth of beneficial bacteria and inhibited the reproduction of harmful bacteria, restoring the balance of the gut microbiota. *Anemarrhena asphodeloides* also triggered the gene expression of peroxidase 4 (PRDX4), promoted pancreatic cell regeneration and restored islet cell function. Studies have confirmed that the gut microbiota and its SCFA metabolites have a significant ability to reduce MetS-induced hypertension. SCFAs have different effects on blood pressure, mainly mediated by different G protein-coupled receptors (GPRs), including olfactory receptor 78 (Olfr78), GPR41, GPR43 and GPR109A [31]. Olfr78 increases blood pressure, whereas GPR41, GPR43 and GPR109A decrease blood pressure. Basal concentrations (0.1–0.9 mmol) of plasma SCFAs activate GPR41/GPR43, which triggers G αi/o-coupled receptors to reduce cyclized adenosine monophosphate (cAMP), inducing vasodilation and lowering blood pressure, whereas higher concentrations (0.9 mmol) of SCFAs activate Olfr78, which triggers Olfr in the olfactory signaling pathway and adenylate cyclase 3 type-induced cAMP production, increasing renin release and leading to hypertension [32]. A corresponding link between the gut microbiota and the pathogenesis of metabolic dysfunction-associated steatotic liver disease (MASLD) has been demonstrated [33], with higher proportions of *Bacteroides/Firmicutes*, increased relative abundance of *Proteus* and *Enterobacteriaceae*, and decreased proportions of *Ruminococcaceae* and *Lactobacillus* in patients with MASLD. Okamura [34] showed that Brazilian green propolis increased the ratio of *Bacteroides/Firmicutes* and the abundance of the genera *Butyricicoccus* and *Acetivibrio*, accelerating the metabolism of glycerolipids, as shown by phylogenetic sequencing using 16S rRNA. The above studies confirm that the gut microbiota has a significant effect on overall energy regulation, whether in the peripheral or central nervous system. Therefore, the gut microbiota is also considered the basis for potential strategies for treating diseases such as obesity, T2DM, IR, and hyperglycemia, which are associated with MetS.

### Adipose dysfunction

Adipose tissue, the endocrine tissue responsible for storing excess lipids, expands by two mechanisms: the enlargement (hypertrophy) of existing fat cells or an increase in the number of fat cells. Excessive visceral adipose tissue (VAT) accumulates in the abdomen (such as the omentum and mesentery) in the form of central obesity or visceral obesity or is distributed throughout the body in the form of lipid droplets wrapped in triglycerides (white adipose tissue (WAT)) [9]. According to data from the World Health Organization, the global obesity rate quadrupled from 1975 to 2016 [35]. In 2016, 39% of adults worldwide (100 million people) were overweight (defined as a body mass index (BMI) ≥ 25 kg/m^2^), whereas the obesity rate among adults reached 13% (BMI ≥ 30 kg/m^2^) (6.501 billion) [36]. By 2030, the number of obese people worldwide is expected to reach an astonishing 1.12 billion, and medical expenses related to obesity and obesity-related diseases are expected to increase by $48 billion to $660 billion annually [35]. Moreover, more than 80% of adults with T2DM are overweight or obese, suggesting that obesity and T2DM are the main drivers of the increase in MetS and CVD patients [37, 38]. As shown in Fig. 1, adipose dysfunction includes many features, such as adipocyte hypertrophy, impaired lipogenesis, reduced efficiency of insulin for lipolysis and low free fatty acid (FFA) uptake, decreased triglyceride synthesis, excessive collagen deposition, impaired remodeling of the extracellular matrix, immune cell infiltration, inflammatory cytokine secretion and remodeling or changes in the structure of blood vessels [9, 39]. An increase in circulating FFA levels leads to a decrease in peripheral glucose utilization and increased gluconeogenesis [40], which further leads to the activation of a variety of inflammatory pathways, the polarization of macrophages to infiltrate visceral and adipose tissues, and the production of inflammatory mediators (such as C-reactive protein). These changes promote the secretion of a variety of cytokines (such as tumor necrosis factor-α (TNF-α), interleukin-6 (IL-6), interleukin-1β (IL-1β), leptin, and adiponectin) and play a key role in regulating systemic metabolism through paracrine and endocrine effects [41]. For example, leptin acts on cognate receptors in the hypothalamus to promote hunger during fasting and to suppress hunger after eating. Lipocalcin enters the blood circulation and acts distally on the liver and muscle to increase sensitivity to insulin, promote fatty acid oxidation, and inhibit lipid accumulation [42]. However, when fat dysfunction occurs in the body, the persistence of inflammation in the body interrupts the normal process of lipolysis, leading to multiple organ damage and dysfunction, causing a series of metabolic dysfunction-related diseases [43–45] (such as T2DM, IR, hypertension, and CVD), and inducing MetS [46, 47].

### Insulin resistance and its underlying mechanisms

Insulin resistance (IR) is a state in which the body and target organs are less sensitive to insulin due to genetic and environmental factors. Insulin fails to reach the normal level for promoting glucose uptake and utilization, and the body compensates by secreting excessive amounts of insulin to maintain the stability of blood glucose [48, 49]. IR is the pathological basis of MetS and T2DM and is the core feature of disease progression. The pathogenesis of IR is complex and involves mainly abnormal insulin signaling, chronic inflammation, oxidative stress, and imbalance of the gut microbiota. IR is related to insulin receptors, the insulin receptor substrate (IRS) family, inositol lipid 3-kinase (PI3K), AMP-activated protein kinase (AMPK)/acetyl-CoA carboxylase (ACC), serine/threonine kinase and tumor necrosis factor-α (TNF-α) [50]. Available genetic and biochemical studies suggest that adipose tissue plays a key role in the development of insulin resistance [51]. Clinical and preclinical studies have shown that in individuals with IR, insulin resistance impairs the response of the body to increasing blood glucose levels, affects glucose metabolism in muscle and the liver, inhibits lipolysis, and mediates the release and uptake of FFAs [52]. As shown in Fig. 1, in terms of lipid metabolism, when adipose tissue is subjected to IR, the ability of insulin to inhibit lipolysis is impaired, leading to an increase in circulating FFA levels, and high concentrations of FFAs promote the synthesis of cholesteryl esters and triglycerides (TGs), leading to an increase in the synthesis of TG-rich very-low-density lipoprotein (VLDL). This increase further activates cholesteryl ester transfer protein (CETP). Moreover, FFAs increase high-density lipoprotein (HDL) clearance, leading to a decrease in HDL [53]. In addition, with respect to glucose metabolism, the damage to body function caused by IR is even more complex. Before understanding the mechanism by which IR affects glucose metabolism, we introduce the concept of “glucose homeostasis”. Under normal circumstances, the glucose level in the blood is maintained in the range of 3.89–6.11 mmol/L, indicating glucose homeostasis [54]. Glucose homeostasis is largely maintained by two processes: glycogen synthesis and gluconeogenesis. Insulin, which is secreted by islet β cells, and glucagon, which is secreted by islet α cells, are the key hormones that regulate blood glucose homeostasis [55], and the ratio of these two hormones is closely related to glucose homeostasis. When humans eat, blood glucose levels rise, and glucose transporter-2 (GLUT2) transports glucose to pancreatic β cells and induces insulin secretion. Insulin promotes glycogen synthesis in muscle and the liver and inhibits gluconeogenesis in the liver and fat through the PI3K/Akt pathway, such that glucose is stored in muscle, the liver and fat in the form of glycogen and fat, thereby maintaining a relatively constant blood glucose level in the body [56]. However, when the body is exposed to IR, the above balance is disrupted. Activation of the PI3K pathway leads to an increase in the synthesis of pyruvate dehydrogenase kinase, type 1 isozymes, and RACα serine/threonine protein kinase; decreased insulin sensitivity in muscle, adipose tissue and the liver; and increased hepatic gluconeogenesis, which in turn stimulate the release of FFAs from excess glucose in vivo. FFAs promote the production of nitric oxide (NO) and endothelial nitric oxide synthase (eNOS) by inhibiting ATP synthesis in muscles and organs [57], leading to the generation and accumulation of reactive oxygen species (ROS), advanced glycation end products (AGEs), and toxic lipids (such as diglycerides (DAG), sphingosine, and long-chain fatty acyl coenzyme A) in the body [9, 58], ultimately leading to glucose homeostasis disorders. Imbalanced glucose homeostasis leads to excessive accumulation of fat tissue in the body, and cytokines secreted by fat tissue accelerate muscle catabolism. Muscle loss further inhibits insulin receptors, reduces the quality of the insulin response, and exacerbates IR and T2DM [59]. Studies have shown that T2DM is often accompanied by other glucose and lipid metabolic disorders, which manifest as decreased insulin secretion and disordered glucose homeostasis and lipid metabolism [49]. For example, nearly 30% − 90% of patients with T2DM have dyslipidemia, 60–85% have hypertension, and approximately 60% of patients with T2DM have MASLD [60]. Thus, IR plays an extremely important role in the pathogenesis of MetS.

### Chronic inflammation and oxidative stress

Multiple studies have shown that the core mechanism underlying the onset and progression of metabolic diseases often involves the interaction between oxidative stress and chronic inflammation within the body [61]. Oxidative stress occurs when the antioxidant defense system of the body is unable to properly eliminate reactive oxygen species (ROS) and reactive nitrogen species, causing their production to far exceed their consumption. As a negative result, accumulated ROS can directly damage cells and their proteins or DNA molecules. These oxidative intermediates are extremely reactive and can attack unsaturated fatty acids in biological membranes, inducing lipid peroxidation and the formation of lipid peroxides. These accumulated peroxides further break down into smaller oxidants such as malondialdehyde (MDA), causing irreversible damage to cells and tissues [61]. As shown in Fig. 1, there are two pathways for intracellular ROS production: nicotinamide adenine dinucleotide phosphate (NADPH) oxidase (NOX) and mitochondrial involvement in metabolism [62]. NOX enzymes are a family of enzymes located on the cell membrane (including NOX1, NOX2, NOX3, NOX4, NOX5, DUOX1, and DUOX2) [61, 62]. In mitochondria, ROS are formed by the oxidation of NADH to NAD^+^ during oxidative phosphorylation [63]. The superoxide anion produced by mitochondria and NOX2, called superoxide dismutase, is rapidly converted to hydrogen peroxide (H_2_O_2_), which plays a vital role in vivo as a signaling molecule. In normal organisms, ROS produced in the body are catabolized by a variety of enzymes (including peroxidase, catalase, glutathione (GSH), and thioredoxin) [61, 64]. However, when the content and activity of oxidases in the body decrease, the production of ROS and oxidative products in the body is far greater than their consumption; thus, these products accumulate and cause oxidative stress.

Obesity is currently recognized as a cause of chronic inflammation, and chronic inflammation is another core mechanism of MetS [65]. As mentioned previously, there is an intrinsic relationship between adipose dysfunction and chronic inflammation. When excessive WAT accumulation leads to obesity, the homeostasis of adipose tissue is imbalanced, and fat metabolism is disrupted. The body stimulates adipocytes to secrete preinflammatory adipokines, resulting in inflammatory cell infiltration around adipose tissue, and the number and function of various immune cells become imbalanced. Chronic inflammation can affect metabolic function in a variety of ways, including dysfunction of adipose tissue, exacerbation of IR, and endocrine disorders [66]. Studies have shown that visceral fat constitutes an inflammatory environment and that macrophages release proinflammatory cytokines, which stimulate the renin–angiotensin–aldosterone system (RAAS), thereby increasing Ang II production [67]. Impaired lipid metabolism increases FFA concentrations, activates Toll-like receptors (TLRs) on endothelial and immune cells, and initiates inflammation and oxidative stress, resulting in the loss of insulin-mediated vasodilation, impaired production of reactive oxygen species and subsequent clearance of nitric oxide, and FFA-induced vasoconstriction and exacerbation of hypertension, causing glucose and lipid metabolism disorders and aggravating MetS [68, 69].

### Epigenetic modifications

Epigenetic modifications refer to a series of changes in gene function that cannot be explained by changes in DNA sequences. Epigenetic changes usually include DNA methylation, histone modification and microRNA expression, which affect functional modification of the genome. As shown in Fig. 1, the literature clearly indicates that epigenetics is highly dynamic and is influenced by various biological and environmental factors, such as aging, nutrition, and physical exercise [70]. There is growing evidence that the benefits of exercise and diet in the prevention and treatment of T2DM, hypertension, IR, and other metabolic diseases may be partly explained by epigenetic factors [71]. Epigenetics can even explain the beneficial effects of genes transmitted through the maternal line on metabolic outcomes in offspring (such as glucose tolerance and glucose clearance) [72]. Epidemiological studies have confirmed that multiple unhealthy lifestyle factors, such as smoking, poor diet, obesity, and lack of physical activity, are present in most patients with chronic diseases (such as CVD, MetS, and degenerative diseases). Nutrition has cross-generational genetic effects, and humans are sensitive to these effects. Therefore, it is widely believed that physical activity and exercise, dietary factors, etc., can regulate gene expression through epigenetic changes and intervene in the occurrence of MetS. For example, under the same dietary intervention, some subjects experience successful weight loss, whereas others experience poor effects or failure. Park et al. [73] reported that “Arylesterase (ARE) and paraoxonase-1 (PON1) activity may be used as markers to predict the response to diet”, suggesting that dietary factors and other epigenetic modifications can have a significant effect on the genomic stability of patients with MetS and the expression of mRNAs and proteins associated with MetS. Similarly, Turcot et al. [74] conducted an experiment in 60 participants with normal weight or obesity and found that as factors such as BMI, obesity, blood pressure, and MetS scores worsened, the methylation levels at certain CpG sites increased. These findings demonstrate that the methylation levels at CpG sites in the CLOCK and PER2 genes were positively correlated with the degree of weight loss in obese women, indicating that some epigenetic changes are associated with the phenotypes of obesity and diabetes. Fujiki et al. [75] reported that, compared with that in control mice, the level of peroxisome proliferator-activated receptor (PPARγ) mRNA expression in the visceral adipose tissue (VAT) of mice with diet-induced obesity (DIO) and db/db mice was lower and that the methylation level of its promoter was greater. These results suggest that DNA methylation levels can serve as biomarkers for MetS and that the DNA methylation-induced decrease in PPARγ expression in VAT may be associated with the pathogenesis of MetS. Heianza et al. [76] conducted an experiment involving 510 overweight and obese individuals who followed a low-calorie diet over a two-year period. At six months, changes in their blood metabolites (trimethylamine oxide, choline, and L-carnitine) were associated with improvements in body weight (BW), waist circumference (WC), body fat composition, fat distribution, and resting energy expenditure (REE). The study revealed that reductions in choline and L-carnitine levels were significantly associated with weight loss, suggesting that they may serve as predictive biomarkers for weight loss and IR treatment response. Therefore, we believe that epigenetic modifications play a nonnegligible role in the pathogenesis of MetS, especially the epigenetic modifications induced by diet, exercise, nutrition, and microbiota/metabolites, which may have potential effects in the treatment of MetS [77].

## Conventional therapeutic interventions

Since the introduction of the ATP III criteria by the Adult Treatment Panel of the National Cholesterol Education Program in 2001 [78], the American Heart Association, the World Heart Federation, the International Society for Atherosclerosis, and the International Association for the Study of Obesity established entirely new diagnostic indicators for MetS in 2009 [79, 80]. Based on these criteria, a key paradigm for the current management of MetS is to address the various conditions involved in MetS simultaneously. Adequately addressing the risks associated with MetS requires lifestyle changes to address the underlying causes, including sedentary lifestyles and physical inactivity, excessive caloric intake, overall poor-quality high-energy–density diets, and excessive or dysfunctional adipose tissue, in addition to targeted pharmacological treatments to ensure optimal control of the major modifiable risk factors [9].

### Lifestyle changes

Lifestyle changes in individuals with MetS focus on behavioral changes such as changed dietary habits, increased exercise, avoidance of a sedentary lifestyle, smoking cessation, moderate alcohol consumption, adequate sleep and stress management [9]. Addressing obesity, especially visceral obesity, is one of the most important treatment options for MetS. From a dietary perspective, key elements include the restriction of carbohydrate and saturated fat intake and increased intake of lean protein, whole grains, fruits, vegetables and omega-3 fatty acids [81]. The Mediterranean diet, the dietary approaches to stop hypertension (DASH) diet, and high-fiber diets all improve multiple aspects of MetS. In terms of increasing exercise, Liang et al. [82] conducted a systematic review of the effects of aerobic exercise on physiological indices in patients with MetS and reported that aerobic exercise can activate muscle groups for a long period and significantly improve body composition as well as blood lipid and blood glucose levels. Extensive studies have shown that smoking is associated with lipid accumulation, endothelial dysfunction, and prothrombotic states. In addition, there is evidence that smoking may exacerbate IR, leading to metabolic and hemodynamic abnormalities that can exacerbate MetS [83]. A clinical trial conducted in Turkey showed that nicotine dependence and anxiety levels in smokers were correlated with MetS parameters [84], and it is therefore reasonable to hypothesize that smoking cessation may alleviate MetS to some extent. A UK study utilizing a large sample and follow-up data from the UK Biobank database of sleep monitoring studies in adult patients in the prediabetic stage revealed a link between poor sleep quality and MetS. Snoring, sleep disorders, and circadian rhythm disorders present in unhealthy sleep states can induce oxidative stress and chronic tissue inflammation, leading to abnormalities in glucose and lipid metabolism and producing a range of symptoms, such as obesity, hyperlipidemia, hypertension, abnormal glucose tolerance, and IR; these results suggest that prolonged periods of insufficient-quality sleep can lead to an increased risk of MetS [85]. However, lifestyle changes require the long-term adherence of patients and attention to factors such as diet, sleep, and psychological states; additionally, increases in exercise and changes in other factors should be made appropriately according to individual circumstances. However, problems such as poor patient compliance and slow results are encountered with these approaches.

### Complex pharmacological interventions

In the management of MetS, lifestyle changes should be combined with targeted drug therapy for optimal efficacy. Currently, drugs targeting obesity, T2DM, hypertension, dyslipidemia, and chronic kidney disease (CKD) are used clinically to reduce cardiovascular-related risk factors and alleviate MetS [9]. For example, Pasanisi et al. [86] studied whether metformin combined with or without Mediterranean dietary intervention could reduce the incidence of MetS in patients through 1,442 volunteers and confirmed that metformin was effective in alleviating MetS, but the effect was dependent mainly on its reduction of glucose levels. Clinical trials of novel antiobesity medications (AOMs) have shown 15 − 20% weight loss in patients, improvements in multiple cardiovascular disease risk factors, and reductions in major cardiovascular events and cardiovascular disease mortality [87]. Common currently available anti-T2DM drugs include high-dose glucagon-like peptide-1 receptor agonists (GLP-1 RAs) and dual/triple-hormone (GLP-1, glucose-dependent insulinotropic polypeptide (GIP) and glucagon) agonists, all of which have been shown to cause varying degrees of weight loss. The only curative treatment for clinical MetS is substantial weight loss, and it is currently treated with multidrug combinations, making it difficult to control the progression of MetS with a single drug. However, given the increasing prevalence of obesity and T2DM, as well as the time and cost of taking medications, these noninvasive interventions are not applicable to the majority of the population. In addition, these drugs do not alter the underlying pathophysiology, as weight is regained after cessation of treatment, and it has not been determined whether the drugs have an effect on the gut microbiota [26]. Furthermore, long-term use of these drugs may cause liver and kidney damage, etc., and there are other adverse reactions. For example, metformin has side effects such as decreased appetite, nausea and vomiting, loose stools, metallic taste and diarrhea [88].

### Bariatric surgery and bariatric management

Interventions initially studied in field of bariatric surgery, such as Roux-en-Y gastric bypass (RYGB), revealed untapped potential for treating abnormal metabolism, including MetS [89]. Historically, RYGB surgery has been the treatment of choice for weight loss when all other medications and dietary options have failed. Bariatric surgery is one of the most effective treatments for morbid obesity, particularly RYGB and vertical gastrectomy (VG), which can result in significant weight loss and improvement in clinical comorbidities, including T2DM and metabolic dysfunction-associated steatohepatitis (MASH) [90, 91]. Many clinical studies have demonstrated that RYGB improves the metabolic health of patients but does not result in changes in patient weight. Clinical approaches (e.g., endoscopy) that have the same effect but are less invasive, including intragastric balloon (IGB) placement, endoscopic sleeve gastroplasty (ESG), duodenojejunal bypass lining (DJBL) or EndoBarrier placement, and duodenal mucosal surface replacement (DMR), are now available. Most studies have shown that the gut microbiota is more diverse after bariatric surgery, e.g., the abundance of the phylum *Actinobacteria* increases and that of *Firmicutes* decreases after RYGB, but the abundance of *Methanobacteriota* and *Escherichia coli* also increases [89]. Gentile et al. evaluated patients who underwent RYGB or VG and reported that, after bariatric surgery, patients showed a significant reduction in the levels of the matrix metalloproteinases MMP-2 and MMP-9, oxidative stress markers, which may be associated with the observed improvements in T2DM and obesity. The authors concluded that bariatric surgery is the most effective therapeutic option for long-term weight loss and improvement in clinical comorbidities (e.g., T2DM) associated with obesity [91, 92]. However, in advanced stages, RYGB is often complicated by postprandial hypoglycemia (PPHG) [93], and these patients are prone to marginal ulcers (MUs) near the anastomotic site, resulting in epigastric pain, nausea, vomiting, or food intolerance [94], which may in turn lead to adverse events and impaired quality of life.

### Emerging therapeutic approaches

In addition to the above therapeutic interventions, the more common clinical treatments are based on functional probiotics and other modalities of cointervention (e.g., lifestyle change plus medication/probiotics or probiotic-assisted drug therapy) [95]. Studies in humans have shown that probiotic supplementation can reduce obesity and enhance metabolic health in individuals, especially when combined with lifestyle interventions for weight loss [96]. Probiotics may affect the development of MetS through their powerful antioxidant properties, modulation of glycolipid absorption and conversion pathways, alleviation of inflammation, and regulation of the gut microbiota [97]. Research has shown that probiotics not only have antipathogenic, anticancer, antiallergic, and proangiogenic activities [98] but can also prevent cardiovascular disease (CVD) [99]. Currently, probiotics are also heavily used in the clinical treatment of MetS. For example, in a clinical study of 44 patients with metastatic cancer, the daily intake of synbiotic yogurt containing the yeast *L. plantarum, L. pentosus*, and *C. marcosianos* markedly reduced IR, systolic blood pressure, and the waist‒to‒hip ratio in the patients [100]. However, long-term dietary guidance and close follow-up are essential for achieving long-term and stable changes in the gut microbiota. Even if patient compliance issues are resolved, a significant proportion of people still do not respond to probiotics [26].

### Systematic intervention and treatment with traditional Chinese medicines

Chinese medicine does not include a complete and specific definition of MetS, and most doctors categorize it as “spleen disease,” “obesity,” “spleen with exuberant damp heat,” etc. According to the evolution of the disease, in TCM, the etiology of MetS is due to polyphagia, lack of exercise, emotional deficiency, congenital deficiency, chronic debility due to aging, etc. According to the theory of TCM combined with the clinical symptoms of patients, MetS can be divided into five types: spleen deficiency with excessive dampness; qi stagnation and blood stasis type; liver and gallbladder dampness heat type; spleen and kidney Yang deficiency type; and simple type. Different treatment methods are used for different patients to reflect the characteristics of TCM syndrome differentiation and treatment. For example, for obese patients, the treatment focus is on invigorating the spleen and digestion; for patients with hypertension, attention is given to suppressing liver hyperactivity and Yang; and for patients with hyperlipidemia, the main treatment direction is to eliminate phlegm and blood stasis. A variety of TCMs and TCM compounds, such as *Poria cocos (Schw.) Wolf, Astragall radix*, *Atractylodes macrocephala Koidz., Alisma plantago-aquatica L, Coptis chinensis Franch.* and *Crataegus pinnatifida Bunge*, have been shown to exert beneficial effects in the treatment of MetS [22, 101, 102]. TCM not only has a rich history in the clinical treatment of MetS but also has the advantages of multiple components with multitarget and multipathway actions [101]. TCMs for MetS are characterized by less gastrointestinal irritation, more precise curative effects, and less damage to the liver and kidneys than Western medicines, as well as broader pathways of action. However, only a single TCM may contain hundreds of ingredients, and explanations of the effects would be difficult to provide in a single article. Polyphenolic compounds are secondary metabolites widely found in a variety of plants. They exhibit different structures and types and are the most common chemical components in TCM [103]. Studies have shown that TCM polyphenols can improve metabolism by regulating the gut microbiota. Most polyphenols in TCMs are also widely used in daily applications such as food additives and pharmaceutical adjuvants and have been gradually accepted by people. For example, resveratrol is widely used in food additives, beverages and cosmetics. Next, this article will introduce different types of TCM polyphenols in detail, including their regulatory effects on the homeostasis of the intestinal flora and their metabolites, and elaborate on their mechanisms and effects in the treatment of MetS.

## Traditional Chinese medicine polyphenols: a promising treatment for metabolic syndrome

### Structure of polyphenols in traditional Chinese medicine

**Fig. 2:**
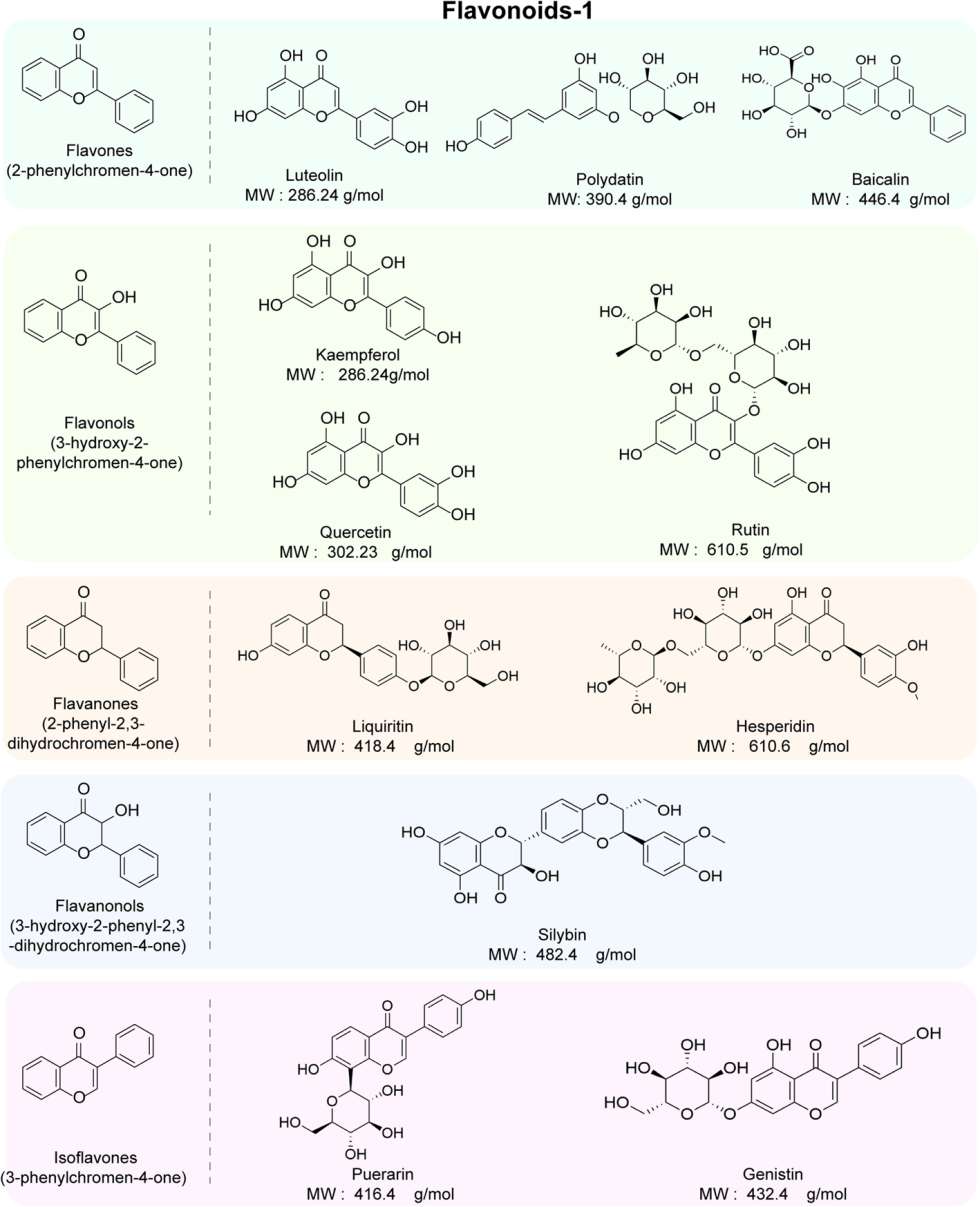
Flavonoid components commonly found in traditional Chinese medicine (TCM) polyphenols, including the parent compounds and international common names of the main categories (e.g., flavones, flavonols, flavanones, flavanonols, and isoflavones). The structural formulas and molecular weights (MWs) of representative components of the 5 categories are also shown

**Fig. 3:**
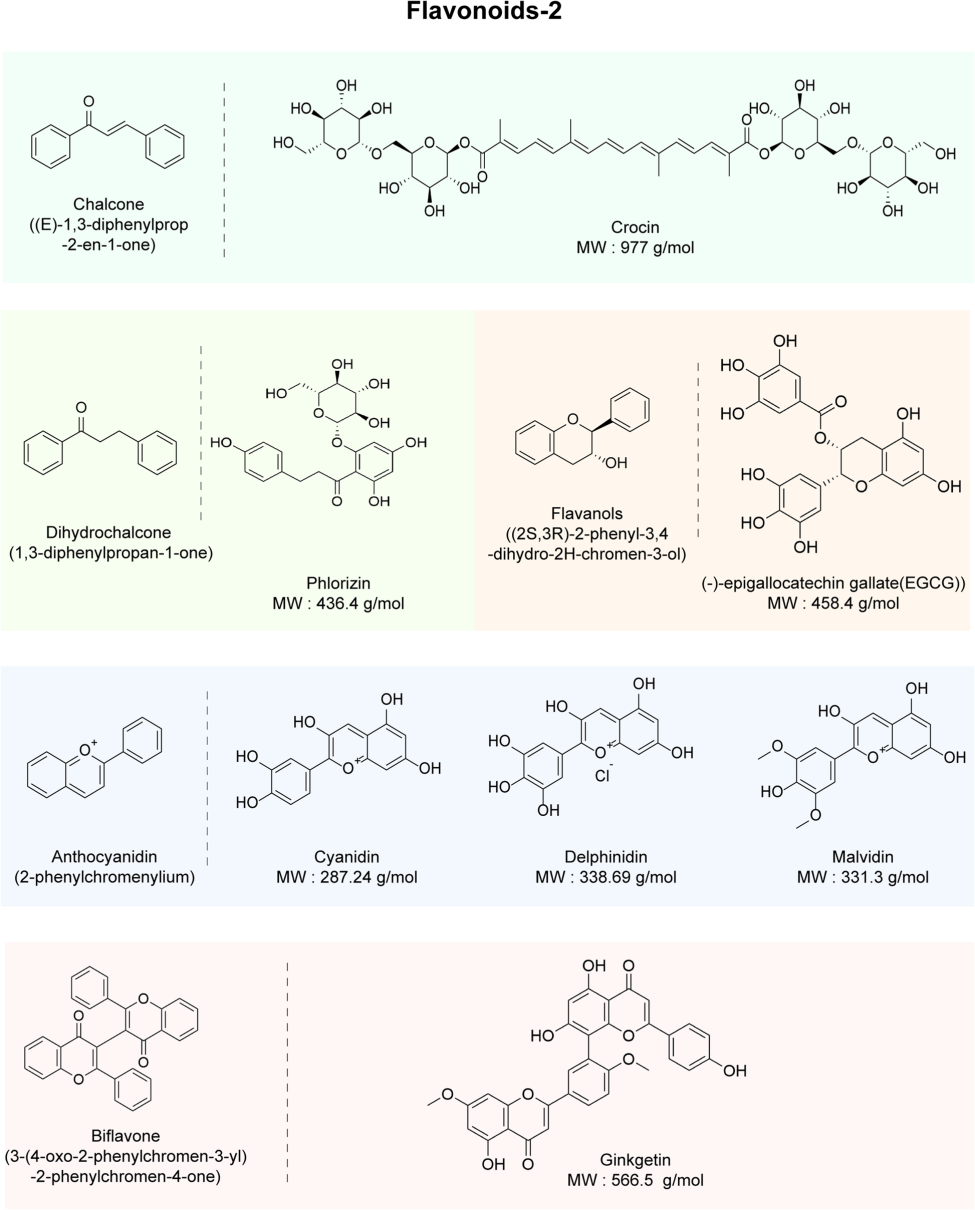
Flavonoid components commonly found in traditional Chinese medicine (TCM) polyphenols (continued from Fig. 2), including the parent compounds and international common names of the main categories (e.g., chalcone, dihydrochalcone, anthocyanidin, and biflavone). The structural formulas and molecular weights (MWs) of representative components of the 4 categories are also included

**Fig. 4:**
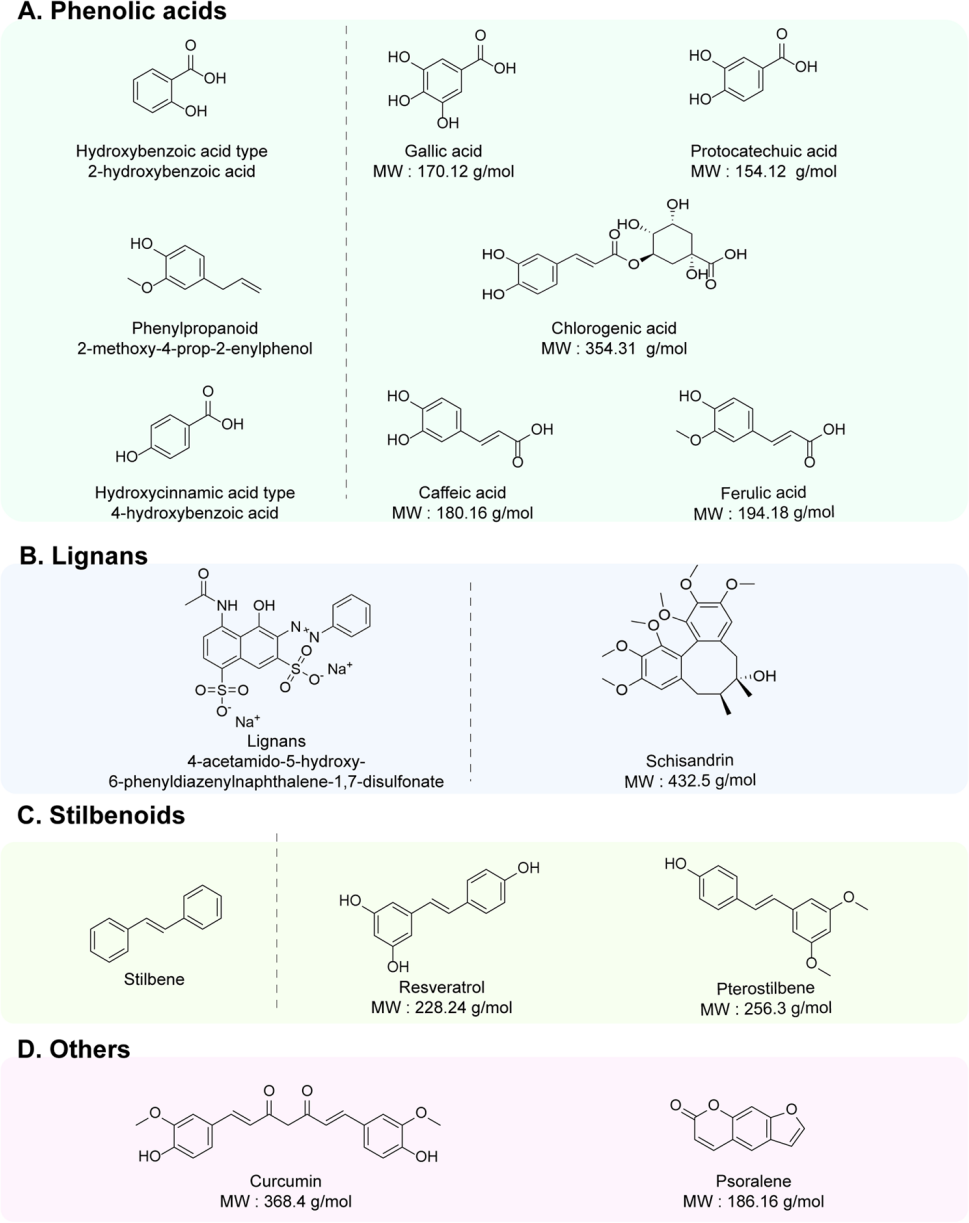
Common nonflavonoid components of traditional Chinese medicine (TCM) polyphenols, including the parent compounds and international common names of the main categories (e.g., phenolic acid, stilbenoid, and lignan). The structural formulas and molecular weights (MWs) of representative components are also shown

Polyphenols are the most common chemical components in TCMs, with flavonoids and phenolic acids accounting for approximately 60% and 30% of the total natural polyphenol content, respectively, making them the most abundant components among polyphenols [104]. Polyphenols are aromatic compounds that contain more than one hydroxyl group, which is directly attached to an aromatic nucleus (benzene or thick benzene ring) [105]. Harborne et al. [106] classified plant polyphenols according to the chemical structure of their carbon atoms. In this work, with reference to this classification, polyphenols were categorized into five groups, including flavonoids and nonflavonoids, on the basis of their chemical structure and origin, where flavonoids include flavones, flavonols, isoflavonoids, and anthocyanins, among others, and nonflavonoids include phenolic acids, lignans, and stilbenoids, among others [104]. Based on the above classification, Figs. 2 and 3 present the structural formula, compound class, representative component and molecular weight of flavonoids in TCM polyphenols, respectively. In addition, Fig. 4 shows relevant information about the nonflavonoid components of TCM polyphenols. In this paper, we present the structure, function, pathways of action, effects on the gut microbiota and benefits of TCM polyphenols to the human body.

### Pharmacological effects of polyphenols in common traditional Chinese medicines

**Fig. 5:**
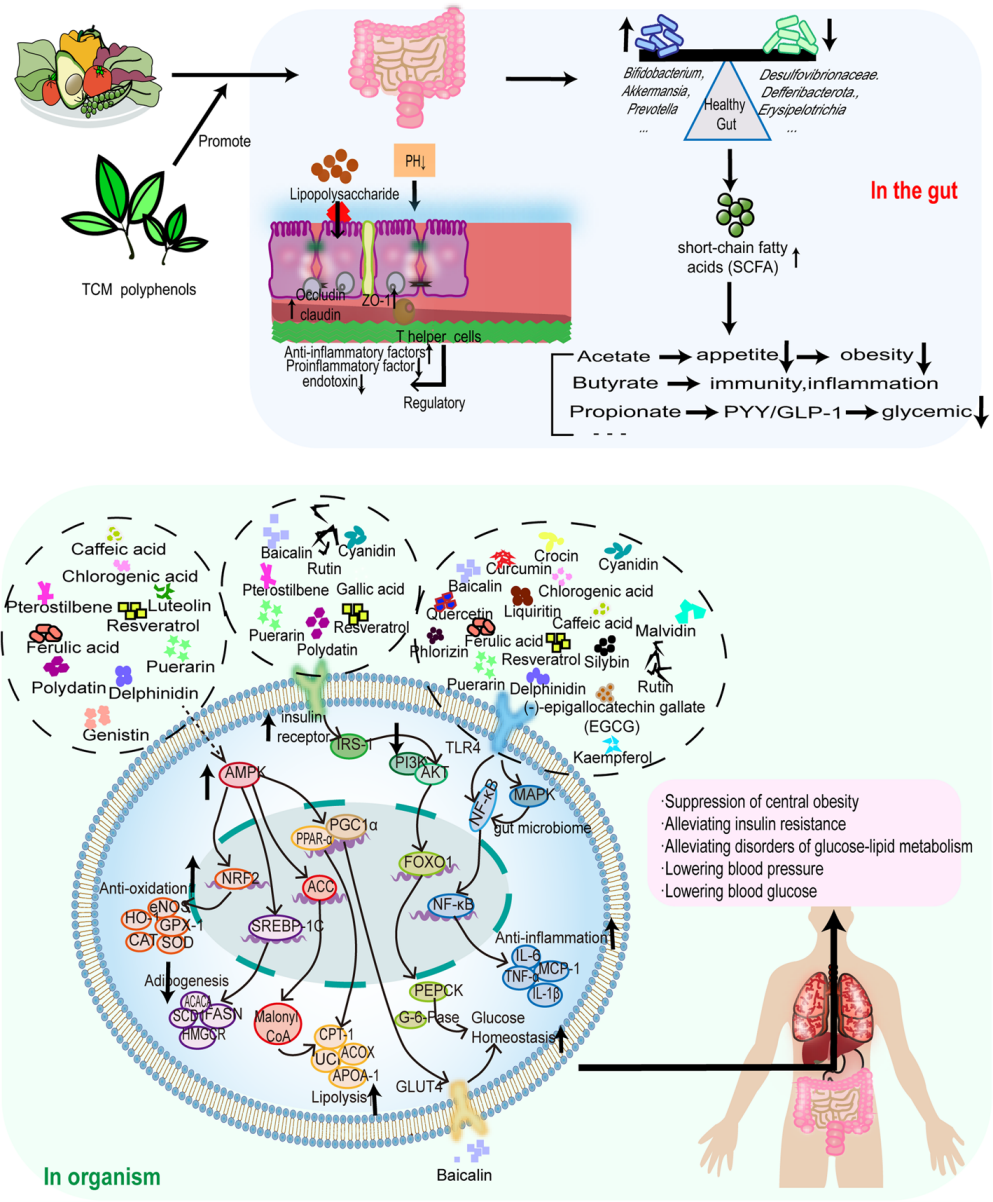
Effects of TCM polyphenols on the intestines and body. After traditional Chinese medicine (TCM) polyphenols enter the intestine, they promote the growth of beneficial bacteria, inhibit the growth of harmful bacteria, and promote the production of short-chain fatty acids (SCFAs). SCFAs in the intestine inhibit the production of lipopolysaccharides, alleviate endotoxemia, maintain pH stability, promote the expression of MUC2 through the NF-κB and MAPK pathways, increase the expression of intestinal tight junction proteins, and enhance intestinal immune function. In vivo, TCM polyphenols produce anti-inflammatory and antioxidant effects, promote adipocyte catabolism, reduce blood pressure, alleviate obesity, and stabilize blood glucose through the AMPK, PI3K/Akt, TLR4/NF-κB, MAPK, and GLUT4 pathways

Research has shown that polyphenolic compounds in TCMs exhibit a variety of pharmacological activities, including anti-inflammatory, antioxidant, antifibrotic, antitumor, hypoglycemic, gastrointestinal digestive, antihypertensive, hypolipidemic, and antiatherosclerotic effects [107, 108]. Epidemiologic research has shown that polyphenol intake is negatively correlated with the incidence of MetS [109]. As shown in Fig. 5, the modulation of MetS by TCM polyphenols may be associated with the peroxisome proliferator-activated receptor (PPAR) signaling pathway, adenylate-mediated protein kinase (AMPK) signaling pathway, farnesoid X receptor (FXR)-small heterodimeric chaperone (SHP) signaling pathway, and phosphatidylinositol-3-kinase (PI3K)-serine/protein kinase B (Akt) signaling pathway, to which the agonist lipopolysaccharide receptor/NF-κB (such as Toll-like receptor 4/NF-κB, TLR4/NF-κB) and glucose transporter protein 4 (GLUT4) are related [110]. The specific effects that each TCM polyphenol exerts through its modulation of the gut microbiota and how it agonizes or inhibits the above receptors and pathways are described next.

#### Modulation of the gut microbiota by flavonoid components

##### Flavones and flavonols

As shown in Fig. 2, flavones are widely distributed in *Angiospermae*, dominated by *Lamiaceae, Scrophulariaceae, Asteraceae, *etc*.* Common flavones include luteolin, polydatin, and baicalin. Flavonols are widely distributed in *Dicotyledoneae*, especially in the flowers and leaves of woody plants, with common flavonols including kaempferol, rutin and quercetin [111].

As shown in Table 1, luteolin is a natural flavonoid that is found mainly in herbs such as *Dracocephalum integrifolium Bunge, Chrysanthemum indicum L., Lonicera japonica Thunb.* and *Perilla frutescens (L.) Britt.,* which show significant antioxidant, anti-inflammatory, antibacterial and antitumor activities [112, 113]. Wang et al. [114] demonstrated that luteolin reduced lipolysis in the epididymal adipose tissue (EAT) of mice fed a high-fat diet (HFD), inhibited lipolytic enzymes in EAT, downregulated 5-HT 2B receptor (Htr2b) and protein kinase G (PKG) expression, and interfered with the activation of SIRT1/FoxO1/AMPKα signaling. In addition, luteolin downregulated the expression of C/EBP-α, PPARγ and SREBP1-c and increased the expression of pACC and pAMPK, preventing adipogenesis and alleviating metabolic disorders. Bolin Li et al. [115] showed by 16S sequencing that luteolin altered the diversity and composition of the gut microbiota in rats exposed to dextran sulfate sodium (DSS), increasing the abundance of *Lactobacillus, Roseburia* and *Butyricoccus*, as the dominant genera in the group, and decreasing the abundance of *Bacteroides, Desulfovibrio, and Coriobacteriaceae*. These findings suggest that improvements in intestinal barrier inflammation may be related to decreases in NF-κB, IL-17 and IL-23 and increases in PPARγ.

**Table 1:** Effects of common flavonoid components of traditional Chinese medicine polyphenols on the gut microbiota and metabolic syndrome

Representative component	Model	Dose	Duration	Changes in the gut microbiota	Mechanism	Reference
Luteolin	mice fed a HFD	17.3 mg/mL luteolin suspension (34.6 mg/kg/d)	2 weeks	*F/B↓; Lactobacillus, Bifidobacterium, Roseburia, Butyricoccus* diversity*↑; Bacteroides, Desulfovibrio, Coriobacteriaceae↓*	↓LPS, TMAO, NF-κB, IL-17, Htr2b, PKG, C/EBP-α, PPARγ, SREBP1-c; ↓hepatocyte TLR4/NF-κB pathway; ↑insulin/IGF-1 signaling in brain cells, SIRT1/FoxO1/AMPKα signaling, pACC, pAMPK	[114, 115]
Polydatin	male C57BL/6 J, 30% fructose-containing drinking water	50 mg/kg/day	10 weeks	SCFA*↑, Allobaculum, Desulfovibrio* spp.*, Muribaculum* spp.*, Bifidobacterium* spp.*↑*	Protein levels of p-AMPK-α (Thr172), CPT-1α, p-ACC (Ser79), p-AKT (Ser473), p-PI3Kp85 (Tyr485), p-IRS1 (Ser307)↑	[116, 117]
Baicalin	6-week-old male C57BL/6 J mice, HFD	400 mg/kg/day	8 weeks	*F/B↑;* SCFA↑*; Lactococcus, Mucispirillum, Parabacteroides, Streptococcus, Ruminococcus, Bacteroides*, *Sutterella↓; Akkermansia, Coprococcus, Ruminococcus, Mucispirillum↑*	↑P38MAPK/PGC-1α/GLUT4 and AKT/AS160/GLUT4 signaling pathways	[118, 119]
	C56BL/6 J male mice, HFD	200 mg/kg/day	15 weeks			
Kaempferol	7-week-old male TSOD and TSNO mice	0.004% or 0.012% in drinking water (5 or 15 mg/kg/day)	6 weeks	*Lachnospiraceae_NK4A136_group, Clostridium, Eubacterium, Ruminococcus, Romboutsia, Faecalibaculum↓; Prevotella, Bacteroides, Akkermansia, Coprococcus↑*	↓TNF-α, IL-6, MCP-1; ↓TLR4/NF-κB pathway; ↑LDL receptor, APOA1	[120, 121]
	8-week-old C57BL/6 J mice, HFD	addition of 0.1% kaempferol to a HFD	16 weeks			
Quercetin	HFS commercial obesogenic diet	30 mg/kg BW/day	4 weeks	*F/B↑, Bifidobacterium, Bacteroides, Clostridium, Lactobacillus, Ruminococcus, Flavobacterium↑; Firmicutes, Erysipelotrichaceae, Bacillus, Eubacterium cylindroides, Erysipelotrichi, Enterococcus, Fusobacterium, Lachnoclostridium, Desulfovibrio, Bilophila, Escherichia coli↓*	Termination of proinflammatory cytokine production (↓IL-17, IL-6, TNF-α)	[122–125]
	87 healthy volunteers	enriched bread with 0.05% of a 1:1 mixture of (-)-epicatechin and quercetin	3 months			
Rutin	6-week-old male C57BL/6 J mice, HFD	100 mg/kg BW, oral gavage	16 weeks	*SCFAs↑; Roseburia, Bacteroides, Alistipes, Parasutterella, Akkermansia↑; Lachnospiraceae, Ruminococcus*, *Erysipelotrichaceae, Faecalibaculum, Proteobacteria, Anaerotruncu↓*	↑TJ expression, remission of endotoxemia; ↓hepatic and adipose tissue TLR/NF-κB signaling	[126, 127]
	fresh feces from 8-week-old male C57BL/6 J mice transplanted into HFD-fed C57BL/6 J mice	100 mg/kg BW	10 weeks			
Liquiritin	C57BL/6 J mice, 30% (wt/vol) fructose	10 or 20 mg/kg	/	*Muribaculaceae, Lachnospiraceae_NK4A136, Turicibacter, Odoribacter, Lactobacillus*, *Bacteroidia, Bacilli↑*	↓lipid accumulation and IR; ↓IKK α/IκBα signaling, ↓NF-κB phosphorylation; ↓MAPKs, improved Treg/Th17 balance	[128–130]
	168-day-old broilers	Penthorum chinense Pursh compound flavonoids, 600 mg/kg, feed	28 days			
Hesperetin	male albino Wistar strain rats, STZ (45 mg/kg BW) soluble in 0.1 M ice-cold citrate buffer (pH 4.5) administered intraperitoneally	40 mg/kg BW	45 days	*↑Ruminococcus_torques_group, Subdoligranulum, Agathobacter, Fusicatenibacter, Blautia, Faecalibacterium, Prevotella;↓ Dorea, Fusicatenibacter* and* Allisonella*	↑SCFA, improved hyperglycemia and hypoinsulinemia, ↑ AMPK	[131–134]
	C57BLKS/Lepr^db^ and INS-1 pancreatic	50, 100, 300 mg/kg/d	6 weeks			
	rat insulinoma INS-1 β cell line, high glucose (HG, 25 mM); simulation of digestion in vivo and fecal fermentation in vitro	10 mg/mL (in vitro simulated digestion), 5 mg/mL (in vitro fecal fermentation)	/			
Silybin	8-week-old male C57BL/6 J mice, HFD	100 or 300 mg/kg/d	8 weeks	*Firmicutes↓, ↓F/B, Lactobacillus* spp*., Lachnoclostridium, Lachnospiraceae_UCG-006* and *Mollicutes_RF9, Alloprevotella, Lactobacillus↓; Bacteroides, Blautia bacterium* and* Akkermansia↑*	↓TNF-α, IL-1β, IL-6; occludin, ZO-1↑, ↑SCFA (acetate, propionate, butyrate production), relief of MASLD	[135, 136]
Puerarin	10-week-old SD female rats	50 or 100 mg/kg/d	14 weeks	*SCFA↑, F/B↓ Clostridia, Melainabacteria, Lachnospiraceae*, *Desulfovibrionaceae*, *Lachnospiraceae_NK4A136_group, Ruminococcaceae_UCG-005, Faecalibacterium, Desulfovibrio, Anaerotruncus, Lachnospiraceae_UCG-001, Erysipelatoclostridium↓; Alloprevotella, Prevotellaceae_UCG-003, Clostridium_sensu_stricto_1, Lactobacillus, Bifidobacterium, Prevotellaceae_NK3B31_group, Akkermansia* and* Ruminococcus↑*	↑PI3K-Akt, AMPK signaling; ↓PPARα/γ; ↓TGF-β1, NF-κB p65	[137–139]
	7-week-old female SD rats, 100 mg/kg TNBS	200 mg/kg	14 days			
Genistin	7-week-old male C57BL/6 J mice, 3% DSS	185 or 740 mg/kg/d	14 days	*F/B↓, Lactobacillus, Lachnospiraceae_NK4A136 group, Allobaculum, Verrucomicrobia, Akkermansia, Lactobacillus rhamnosus, Faecalibacterium prausnitzii↑; Helicobacter, Proteobacteria, Bacteroides fragilis, Lactococcus lactis* subsp. *lactis,* and* Slackia equolifaciens↓*	↓AMPK, PPARα, PPARγ, NF-κB, ↓MAPK; reduced expression of cytokines (TNF-α, IL-6 and IL-1β); antioxidative stress (ROS↓)	[140, 141]
Crocin	7-week-old male C57BL/6 J wild-type mice and LDLR mice (atherogenic high-fat/cholesterol diet)	20 mg or 40 mg/kg/d	8 weeks	*F/B↓, Bacilli, Lactobacillale↑, Erysipelotrichia*, *Parabacteroides ↓*	↑hepatic T-AOC, ↑Nrf2 and Keap1 degradation, ↑HO-1; PPARγ, AMPK, ↓IL-6, TNF-α, NLRP3 inflammatory vesicles, ↓TLR4	[142–144]
	male Wistar rats, 3% NaCl and 10% fructose	40 mg/kg/d	8 weeks			
Phlorizin	6- to 8-week-old male SD rats, high-fat/alcohol diet	200 or 600 mg/kg BW	12 weeks	*Aspergillus, Prevotella, Ruminococcus, Lactobacillus, Akkermansia*, *Bacteroides ↑, Desulfovibrio*, *Allobaculum↓,*	↑SCFAs, ↑AMPK, PPARα, NF-кB, caspase-9; ↓TNF-α, IL-6, MCP-1	[145, 146]
	6-week-old male db/db mice	20 mg/kg BW	10 weeks			
EGCG	6-week-old C57BL/6 J male mice, HFD	25 mg/kg BW	4 months	*F/B↓, Lactobacilli, Desulfovibrio, Alistipes, Ruminococcus, Lachnospiraceae↓, Clostridium cluster IV, Bacteroidetes, F. prausnitzii, Akkermansia, Coprococcus, Prevotella, Bifidobacterium, Actinomyces, Adlercreutzia* spp.,* Allobaculum ↑*	↑NF-κB p65, ↓oxidative stress, NR3C1	[147–150]
	3-week-old male Swiss mice	50 mg/kg BW per day	30/60 days			
	65 adults aged 18–30 years with a BMI between 19 and 25 kg/m^2^ and SD rats	200/100/50/20 mg/kg	/			
Cyanidin	51,529 male health professionals aged 40–75 years; 121,701 female nurses aged 30–55 years; 116,686 young female nurses aged 25–42 years	/	/	*Blautia, Lachnospiraceae, Rikenella*, *Desulfovibrio↓; Ruminococcus, Muribaculaceae, Bacteroides, Helicobacter, Akkermansia muciniphila*↑	↓TLR4/NF-κB signaling (TNF-α, MCP-1, IL-6); ↓ROS; ↑Akt, AMPK signaling	[151–154]
	25 healthy volunteers aged 19–35 years, body mass index > 21 and < 29.9 kg/m^2^	4 g CDRE	3 days			
Delphinidin	8-week-old male C57BL/6 J mice	5 mg/kg/d or 15 mg/kg/d	4/8 weeks	*Bifidobacterium* spp.*, Lactobacillus-Enterococcus, Akkermansia muciniphila, Bifidobacterium, Parabacteroides, Lachnospiraceae↑; Clostridium, Escherichia, Erysipelotrichaceae, Staphylococcus↓*	Reversed metabolic endotoxemia, ↓hepatic TLR2, TLR4, ERK1/2, AP-1 and HIF-1; MCP-1, TNF-α, iNOS, NADPH oxidases (NOX1, NOX4)↓	[152, 155–157]
	(STZ)-induced male BALB/c mice	100 mg/ml	8 weeks			
	5-week-old male C57BL/6 J mice	50 mg cyadinin (28.9 mg) + delphinidin (21.1 mg) per kg BW	4 weeks			
	25 healthy volunteers aged 19–35 years, body mass index > 21 and < 29.9 kg/m^2^	4 g CDRE	3 days			
Malvidin	one healthy female donor (24 years old)	Anaerobic incubation of intestinal microbiota in vitro (600, 300, 100 μmol/L)	/	*F/B↓, Enterococcus, Faecalibaculum, Lactobacillus, Prevotella, Bifidobacterium, Ruminococcus↑, Escherichia-Shigella, Streptococcus↓*	↑TFEB, LAMP1, ↑Nrf2/ARE, SOD, GPx, CAT, increased lipid deposition	[158, 159]
Ginkgetin	male SD rats	25 mg/kg/d, 50 mg/kg/d, 100 mg/kg/d, intraperitoneally injected with vitamin D3 at 600,000 IU/kg and chow diet	8 weeks	*Bifidobacterium, Lactobacillus, Enterococcus↑*	↓mRNA and protein expression of MMP-2 and MMP-9 in the aorta; ↓STAT5, PPARγ, C/EBPα	[160–162]

*HFD* high-fat diet, *LPS* lipopolysaccharide, *TMAO* trimethylamine N-oxide, *TLR4* Toll-like receptor 4, *NF-κB* nuclear factor kappa-B, *IGF-1* insulin-like growth factor 1, *AMPK* adenosine 5’-monophosphate (AMP)-activated protein kinase, *Thr172* phospho-AMPK alpha 2, *CPT-1α* carnitine palmitoyltransferase1α, *p-ACC (Ser79)* phospho-acetyl-CoA carboxylase (Ser79), *p-AKT (Ser473)* phospho-AKT1 (Ser473), *p-PI3Kp85 (Tyr485)* phospho-PI3 kinase p85 (Tyr458), *p-IRS1 (Ser307)* phospho-IRS1 (Ser307), *MAPK* mitogen-activated protein kinase, *PGC-1α* peroxisome proliferator-activated receptor-γ coactivator-1α, *GLUT4* glucose transporter type 4, *AKT* protein kinase B, *AS160* AKT substrate of 160 ku, *TNF-α* tumor necrosis factor-α, *IL-6* interleukin-6, *MCP-1* monocyte chemoattractant protein 1, *LDL* low-density lipoprotein cholesterol, *APOA1* apolipoprotein A1 gene expression, *HFS* high fat and high fructose, *BW* body weight, *F/B Firmicutes/Bacteroidetes*, *SCFAs* short-chain fatty acids, *TJs* tight junctions, *IR* insulin resistance, *IKK α* inhibitor of kappa B kinase, *IκBα* I-kappa-B-alpha, *Treg* regulatory T cell, *Th17* T helper cell 17, *STZ* streptozotocin, *INS-1* rat islet cell tumor cells, *SD* Sprague–Dawley, *ZO-1* zona occludens 1, *MASLD* metabolic dysfunction-associated steatotic liver disease, *TNBS* 2,4,6-trinitrobenzenesulfonic acid sol, *PI3K* phosphatidylinositol 3-kinase, *PPAR* peroxisome proliferator-activated receptor, *TGF-β1* transforming growth factor beta 1, *ROS* reactive oxygen species, *T-AOC* total antioxidant capacity, *Nrf2* erythroid-derived 2, *Keap1* kelch-like ECH-associated protein 1, *HO-1* heme oxygenase 1, *NLRP3* NOD-like receptor family pyrin domain containing 3, *Caspase* cysteinyl aspartate specific proteinase, *BMI* body mass index, *NR3C1* nuclear receptor subfamily 3 group C member 1, *ERK1/2* extracellular-regulated protein kinases 1/2, *AP-1* activator protein 1, *HIF-1* hypoxia inducible factor 1, *iNOS* inducible nitric oxide synthase, *NADPH* nicotinamide adenine dinucleotide phosphate, *NOX* NADPH-oxidase, *TFEB* T cell transcription factor EB, *LAMP1* lysosomal-associated membrane protein 1, *ARE* AU-rich element, *SOD* superoxide dismutase, *GPx* glutathione peroxidase, *CAT* catalase, *MMP* matrix metalloproteinase, *STAT* signal transducer and activator of transcription, *C/EBPα* enhancer binding protein alpha

Polydatin, extracted from *Polygonum L. Polygonum cuspidatum Sieb. et Zucc.,* is a natural precursor compound that exists in the form of a glycoside of resveratrol. It exhibits significant pharmacological activities, including antibacterial, anti-inflammatory, antioxidant, vasodilatory, antithrombotic, promicrocirculatory, and antishock effects [163, 164]. Polydatin may act as a potent vascular endothelial protector in diabetes, and acetylcholine-induced vasodilation and endothelial disruption due to high glucose can be restored by polydatin in a dose-dependent manner [116]. Zhao et al. [117]. demonstrated that the administration of 50 mg/kg/day polydatin to 5-week-old C57BL/6 J MASLD model mice significantly increased the diversity of intestinal *Lactococcus, Allobaculum, Desulfovibrio* spp. and *Bifidobacterium* spp.; significantly increased the fecal levels of valeric and hexanoic acids; increased the p-AMPK-α (Thr172), CPT-1α, p-ACC (Ser79), p-AKT (Ser473), p-PI3Kp85 (Tyr485), and p-IRS1 (Ser307) protein levels; promoted endothelial NO synthase (eNOS) expression; increased eNOS activity; and decreased inducible NOS (iNOS) levels through the activation of PPARα and PPARγ, ultimately leading to increased NO release. These effects promoted vascular endothelial cell (VEC) function in diastole and improved IR, disorders of glucose-lipid metabolism and MASLD [116].

As shown in Fig. 2, baicalin was extracted from the dried root of *Dicotyledoneae Lamiaceae Scutellariabaicalensis Georgi* to obtain the flavonoid constituents [165], and research has shown that baicalin has good antitumor, antiviral, anti-inflammatory, and myocardial cell protective properties [166]. Lin et al. [118] demonstrated that baicalin reversed HFD-induced alterations in seven metabolites, as well as metabolic pathways responsive to baicalin intervention, including the citric acid cycle; alanine, aspartate, and glutamate metabolism; glycerophospholipid metabolism; and aminoacyl-tRNA biosynthesis. Baicalin increases the relative abundance of *Bacteroidetes*; elevates the *F/B* ratio; decreases the abundance of *Lactococcus, Mucispirillum, Parabacteroides, Streptococcus, Ruminococcus, Bacteroides* and *Sutterella*; and increases the abundance of *Akkermansia, Coprococcus, Ruminococcus,* and *Mucispirillum*. These effects promote SCFA production, protect the intestines system, and regulate multiple metabolic and immune processes in organisms through activation of the P38MAPK/PGC-1α/GLUT4 and Akt/AS160/GLUT4 signaling pathways, suggesting that baicalin may be a promising therapeutic agent for metabolic disorders [118, 119].

Kaempferol is a flavonoid compound widely found in *Kaempferia galanga L., Nelumbinis folium, Croci stigma, Astragali radix*, and other TCMs; it exhibits antioxidant, antitumor, and anti-inflammatory effects, as well as beneficial effects on diabetes, atherosclerosis, and osteoporosis [167]. Bian et al. [120] reported that supplementing mice fed a HFD with kaempferol effectively alleviated MetS symptoms such as obesity, fat accumulation, glucose tolerance abnormalities, and lipid inflammation caused by the HFD. These effects may be related to kaempferol completely blocking the HFD-induced increase in *Firmicutes* and decrease in *Bacteroidetes*; increasing the ratio of *Firmicutes/Bacteroidetes*; inhibiting *Lachnospiraceae_NK4A136_group, Clostridium, Eubacterium, Ruminococcus, Romboutsia,* and *Faecalibaculum* growth in HFD-fed mice; promoting *Prevotella*, *Bacteroides, Akkermansia* and *Coprococcus* growth; and regulating insulin sensitivity by downregulating serum TNF-α, IL-6 and MCP-1 and TLR4/NF-κB signaling, as well as increasing LDL receptor and ApoA1 gene expression to improve intestinal barrier integrity and inhibit intestinal inflammation [120, 121].

Quercetin is a flavonoid widely found in nature in *Sophorae flos, Cichorii herba Cichorii radix, Chrysanthemi flos, Lycii fructus, Mori folium* and *Astragali radix.* Numerous studies have shown that the powerful antioxidant properties of quercetin can help prevent cancer, delay aging, and protect the cardiovascular, hepatic and nervous systems [168]. Studies have demonstrated that quercetin supplementation improves intestinal barrier function, maintains intestinal barrier stability, reduces serum diamine oxidase activity and serum D-lactate levels [169], reduces lipid peroxidation in serum and liver tissue, and improves free radical and ROS scavenging [170]. A randomized controlled trial involving 156 volunteers revealed that after consuming bread containing 0.05% (-)-epicatechin and quercetin (1:1 mixture ratio) for 3 consecutive months, total cholesterol (TC), low-density lipoprotein cholesterol (LDL), triglyceride (TG), and fasting blood glucose levels significantly decreased [122]. U Etxeberria [123] revealed that in Wistar rats fed a high-fat, high-sucrose diet (HFS), trans-resveratrol and quercetin administration counteracted the dysbiosis of the gut microbiota induced by the HFS diet by attenuating the *F/B* ratio; increasing the abundance of *Bifidobacterium, Bacteroides, Clostridium, Lactobacillus, Ruminococcus, Flavobacterium,* etc.; decreasing the abundance of *Firmicutes, Erysipelotrichaceae, Bacillus, Eubacterium cylindroides, Erysipelotrichi, Enterococcus, Fusobacterium, Lachnoclostridium,* etc.; and reducing inflammation and IR. In addition, quercetin downregulated the abundance of the genera *Desulfovibrio* and *Bilophila*; decreased the abundance of *Escherichia coli*; promoted intestinal homeostasis; terminated the production of proinflammatory cytokines, including IL-17, IL-6, and TNF-α; and increased the production of IL-10 in colonic tissue [124, 125].

Rutin is derived from TCMs such as *Flos Sophorae Immaturus, Mori folium, Cirsii herba,* and other TCMs, as well as other fruits and vegetables, and is a common component of flavonoids that are widely produced [171]. Rutin exhibits diverse pharmacological properties, including anticancer, anti-inflammatory, neuroprotective, and antioxidant effects [172, 173]. Yan et al. [126] supplemented 6-week-old C57BL/6 J male mice fed a HFD with rutin and confirmed that rutin not only was effective in reducing insulin resistance but also exerted a favorable effect on the intestinal barrier. Rutin completely blocked the HFD-induced increase in the ratio of sequestered bacteria/bacteroidetes, and the gut microbiota of rutin-treated mice presented a variable increase in the abundance of the genera *Roseburia, Bacteroides, Alistipes, Parasutterella*, and *Akkermansia* and a decrease in the abundance of *Lachnospiraceae, Ruminococcus*, *Erysipelotrichaceae*, *Faecalibaculum, Proteobacteria* and *Anaerotruncu*. In addition, rutin upregulates the expression of tight junction (TJ) proteins, reduces endotoxemia, inhibits hepatic and adipose TLR/NF-κB signaling, and maintains intestinal barrier stability, thus improving the systemic inflammatory response to effectively inhibit weight gain, adiposity, metabolic disorders and chronic inflammation [126, 127].

##### Flavanones and flavanonols

As shown in Fig. 2, flavanones are widely distributed, especially in the angiosperms *Rosaceae Juss., Rutaceae Juss., Fabaceae Lindl.,* and *Asteraceae Bercht*. Flavanonols are found mainly in citrus fruits; liquiritin is a common flavonoid in TCMs, and silybin is a flavanonol [174]. Liquiritin is a flavanone that can be obtained from *Glycyrrhiza uralensis Fisch., Glycyrrhiza inflata Bat.* or *Glycyrrhiza glabra L.* and showed a wide range of pharmacological activities, including antidepressant, antioxidant, anti-inflammatory, antiviral, and antitumor activities, as well as protective effects on cardiomyocytes [128, 175]. Zhang et al. [128] demonstrated that the effective alleviation of fructose-induced lipid accumulation and IR by liquiritin was associated with inhibition of the IKKα/IκBα signaling pathway, which significantly reduced inflammatory factor release and NF-κB phosphorylation, in turn downregulating mitogen-activated protein kinase (MAPK) expression, in mice fed a high-fructose diet. In addition, the major bacterial taxa whose abundance is altered by liquiritin include *Muribaculaceae, Lachnospiraceae_NK4A136, Turicibacter, Odoribacter, Lactobacillus*, *Bacteroidia* and *Bacilli,* most of which are described as beneficial bacteria, along with *Lachnospiraceae, Turicibacter, Romboutsia, Saccharimonas* and *Odoribacter*, whose relative abundance is directly related to the concentration of SCFAs. Additionally, glycyrrhizin improved the Treg/Th17 balance during the progression of colitis and was effective in relieving ulcerative colitis [129, 130].

Hesperidin is widely found in *Leguminosae, Faboideae, Labiatae,* and *Rutaceae*, among others. In TCM, it is commonly found in *Citri Reticulatae Pericarpium,* and *Aurantii Fructus* and exerts antitumor, antioxidant, anti-inflammatory, antiaging and antiatherosclerotic effects [176–178]. By using STZ-induced Wistar strain rats, Jayaraman et al. [131] reported that hesperidin reduced plasma insulin levels and ameliorated hepatic, renal, and β-islet lesions in diabetic rats. By using in vivo digestion simulations and in vitro fecal fermentation, Wu et al. [132] found that hesperidin can increase the relative abundance of *Ruminococcus_torques_group, Subdoligranulum, Agathobacter, Fusicatenibacter*, *Blautia, Faecalibacterium,* and *Prevotella,* thereby promoting the production of SCFAs and increasing the bioavailability of hesperidin. In addition, Wang et al. [133] used db/db mice to demonstrate that hesperidin can protect pancreatic β cells, reduce pancreatic damage in db/db mice, prevent pancreatic cell death, and improve IR in db/db mice. Moreover, in the INS-1 β-cell line, hesperidin lowered blood glucose and improved islet injury by activating the AMPK pathway.

Silybin, representing a class of active ingredients extracted from the dried fruit of *Silybum marianum (L.) Gaertn*, has a long history of efficacy in the treatment of metabolic dysfunction-associated steatohepatitis (MASLD) [134]. Silybin exerts significant antioxidant, anti-inflammatory, hepatoprotective, antifibrotic, antitumor, antiviral, anticancer, hypoglycemic, antineurodegenerative, and immunomodulatory effects [134, 179, 180]. Li et al. [135] examined changes in the gut microbiota and its metabolites in mice fed a HFD after 10 weeks of silybin administration. They reported that silybin led to a reduction in the abundance of *Firmicutes*; a reversal of the *F/B* ratio; a significant decrease in the abundance of *Lactobacillus, Lachnoclostridium, Lachnospiraceae_UCG-006,* and *Mollicutes_RF9*; and a significant reduction in the abundance of *Bacteroides, Blautia bacterium*, and *Akkermansia*. In addition, the production of acetic acid, propionic acid and butyric acid was increased in the silybin-treated group, whereas the production of formic acid and the conversion of cytotoxic secondary metabolites were decreased. Silybin treatment tended to decrease the levels of TNF-α, IL-1β, and IL-6, which induced a significant increase in the levels of the tight junction proteins occludin and ZO-1, which could be effective in treating intestinal barrier inflammation and alleviating the effects of MASLD [136].

##### Isoflavones

As shown in Fig. 2, the isoflavone nucleus is 3-phenylchromanone, which is mainly distributed in *Angiospermae*, *Papilionoideae* and *Iridaceae*. As shown in Table 1, puerarin is the main medicinal component of *Puerariae thomsonii radix* and *Puerariae lobatae radix*, which can regulate inflammation, lipids, blood glucose, and immunity; protect hepatic and cardiovascular functions; and exert significant therapeutic effects, especially on CVD [181, 182]. Puerarin may promote SCFA secretion by regulating changes in the gut microbiota. Specifically, it increases the *F/B* ratio and decreases the abundance of *Clostridia, Melainabacteria, Lachnospiracea*e, *Desulfovibrionaceae*, *Lachnospiraceae_NK4A136_group, Ruminococcaceae_UCG-005, Faecalibacterium, Desulfovibrio, Anaerotruncus, Lachnospiraceae_UCG-001* and *Erysipelatoclostridium* while increasing the abundance of *Alloprevotella, Prevotellaceae_UCG-003, Clostridium_sensu_stricto_1, Lactobacillus, Bifidobacterium*, *Prevotellaceae_NK3B31_group, Akkermansia* and *Ruminococcus*. Additionally, Puerarin increases mucin (Muc2 and Muc4) levels and cuprocyte numbers and promotes intestinal barrier stabilization [137, 138]. Puerarin also inhibits the release and transport of glucose and FFAs, acts on the PI3K-Akt and AMPK signaling pathways to reduce glucose and fatty acid synthesis, downregulates the expression of PPARα/γ, inhibits the expression of TGF-β1 and NF-κB p65 to exert antifibrotic effects, and improves insulin secretion and sensitivity [139].

Genistin, also known as goldfinch isoflavone, is a form of genistein, and its chemical structure is similar to that of endogenous estradiol, exhibiting similar estrogen receptor binding and estrogen-like effects. Genistin is widely found in legumes such as *Eucommiae cortex, Puerariae lobatae radix, Spatholobi caulis,* and *Sophorae tonkinensis radix et rhizoma*, as well as other herbs [183]. Genistein is known to prevent osteoporosis, protect the cardiovascular system, and have antitumor, antioxidant, hepatoprotective and antimicrobial activities [184, 185]. Lucía Vázquez et al. [140] determined the minimum inhibitory concentrations (MICs) of isoflavone glycosides and strychnine against 37 bacterial strains using a broth microdilution assay and reported that genistein decreased the abundance of *Bacteroides fragilis*, *Lactococcus lactis* subsp. *lactis* and *Slackia equolifaciens* and increased the abundance of *Lactobacillus rhamnosus* and *Faecalibacterium prausnitzii,* demonstrating that isoflavone-derived compounds can alter the populations of key bacterial species in the gut. Genistein significantly decreased the *F/B* ratio; significantly increased the abundance of *Lactobacillus, Lachnospiraceae_NK4A136_group*, *Allobaculum*, *Verrucomicrobia* and *Akkermansia*; and significantly reduced the abundance of *Helicobacter* in HFD-fed mice. Furthermore, this compound promoted the production of SCFAs; effectively inhibited the expression of the energy metabolism-related proteins AMPK, PPARα and PPARγ; downregulated the expression of NF-κB pathway proteins; inhibited the release of inflammatory factors; and ultimately prevented obesity and its complications [141].

##### Chalcone and dihydrochalcones

As shown in Fig. 3, chalcone is more widely distributed in *Asteraceae* and *Fabaceae Lindl* plants. Chalcone is a benzaldehyde acetophenone analog, and its 2′-hydroxy derivative is an isomer of dihydroflavone [186]. As shown in Table 1, crocin (also known as saffron glycoside) is the active ingredient in *Croci stigma* and is widely used for its pharmacological effects, including anti-inflammatory, antioxidant, antiviral, antitumor, immune-boosting, and memory-improving effects [187, 188]. The pathological results of LDL mice fed an atherogenic HFD and treated with crocin by gavage for 8 weeks revealed that crocin markedly reduced atherosclerotic plaques. This change occurred with an increase in the abundance of *Bacilli* and *Lactobacillale*, a decrease in the abundance of *Erysipelotrichia* and *Parabacteroides*, and a decrease in the *F/B* ratio [142]. Crocin intervention attenuated CD68, enhanced antioxidant effects by increasing hepatic T-AOC levels and Nrf2 pathway activation as well as Keap1 degradation, enhanced HO-1 expression, reduced the entry of bacterial LPS into the circulation, improved intestinal permeability by increasing the expression of tight junction proteins, which are critical for the function of the epithelial barrier, and alleviated NLRP3 inflammatory vesicles and TLR4 signaling by inhibiting the systemic inflammatory response [142, 143]. In addition, Algandaby [144] demonstrated that crocin significantly increased serum PPARγ levels, activated AMPK signaling, increased β oxidation of fatty acids and inhibited adipogenesis in rats, effectively alleviating elevated blood IL-6 and TNF-α levels and mitigating inflammation.

Dihydrochalcones are flavonoids that are uncommon in nature, and phlorizin, the main active ingredient of the Tibetan medicine *Malus toringoides (Rehd.) Hughes* and *Lithocarpus polystachyus*, belongs to this category [189]. Phlorizin shows various pharmacological activities, such as antioxidant, hypoglycemic, anti-inflammatory, and antitumor activities. As a precursor to phloretin, phlorizin is the first polyphenol identified as a sodium‒glucose cotransporter protein (SGLT) located in the small intestinal mucosa (SGLT1) and the proximal renal tubule (SGLT2); as a competitive inhibitor, it improves hyperglycemia through the blockade of renal glucose reabsorption and intestinal glucose absorption [145]. Phlorizin significantly increases the abundance of *Aspergillus, Prevotella, Ruminococcus, Lactobacillus, Akkermansia muciniphila* and *Bacteroides* while decreasing the abundance of *Desulfovibrio* and *Allobaculum,* leading to an important increase in the intestinal SCFA content and resulting in a marked reduction in the serum LPS levels in diabetic mice. Additionally, phlorizin has shown significant anti-inflammatory effects in obese mice, notably by lowering the levels of proinflammatory factors, such as TNF-α, IL-6, and MCP-1, which alleviated insulin resistance and improved MetS [145, 146].

##### Flavanols

As shown in Fig. 3, derivatives of flavan-3-ols are known as catechins, which are widely distributed in plants and are found mainly in woody plants containing ellagitannins [190]. (-)-Epigallocatechin gallate (EGCG) has excellent antioxidant activity and can effectively prevent various diseases caused by oxidative stress (CVD, diabetes, and cancer). It is found mainly in green tea and has the ability to lower cholesterol and protect the heart [191–193]. In one study including 65 adults aged 18 to 30 years with a BMI between 19 and 25 kg/m^2^, participants consumed potato chips containing 200 mg/100 mg/50 mg TP and 12.6 μg/kg BW acrylamide. The results showed that EGCG significantly improved the disorders in glucose and lipid metabolism, the tricarboxylic acid cycle, and phenylalanine metabolism caused by acrylamide intake [147]. As shown in Table 1, the abundance of *Lactobacillus, Desulfovibrio, Alistipes, Ruminococcus,* and *Lachnospiraceae* decreased with EGCG intervention, whereas the abundance of *Coprococcus, Prevotella, Bifidobacterium, Actinomyces, Adlercreutzia* spp.*, F. prausnitzii, Akkermansia muciniphila,* and *Allobaculum* increased [148]. EGCG-containing diets increased the phosphorylation of the p65 subunit of the NF-κB complex in mesenteric adipose tissue, reduced adipose tissue deposits, lowered body weight in mice, and decreased TG and HDL-C production in fat [149]. In addition, EGCG can protect pancreatic β cells from excessive autophagy induced by NR3C1 enhancement by inhibiting FTO-stimulated oxidative stress, thus achieving hypoglycemic and pancreatic protective effects [150].

##### Anthocyanidins

As shown in Fig. 3, anthocyanidins are pigments that make the flowers, fruits, leaves and stems of plants blue, purple and red, among other colors. Anthocyanidins are widely distributed in *Angiospermae*, especially cornflower, with the most common including delphinidin, pelargonidin and glycosides [194]. As shown in Table 1, cyanidins are colored glycosides produced by the hydrolysis of anthocyanins, which are found mainly in *Perillae folium*. They have various biological activities, including antioxidant and antitumor activities, beneficial effects on vision, protective effects on the cardiovascular system, regulatory effects on blood sugar, and antibacterial, anti-inflammatory, and immunoregulatory activities [194, 195]. In a clinical study, Bertoia et al. [151] found that consuming foods rich in flavonols, flavan-3-ols, anthocyanins, and flavonoid polymers may help prevent obesity in adolescents and maintain weight in adulthood. In 25 healthy volunteers aged 19–35 years with BMI > 21 and < 29.9 kg/m^2^, the administration of 4 g of CDRE (cyanidin- and delphinidin-rich extract) plus 1 g of AC (150 mg of bilberry extract: 230 mg of black currant extract: 620 mg of black rice extract) and 3 g of maltodextrin mixture improved postprandial metabolic dysregulation, inflammation, oxidative reduction and inflammation following the consumption of a high-fat meal [152]. Cyanidin works by regulating the gut microbiota through SCFA-producing bacteria (e.g., *Ruminococcus, Ruminococcus, Muribaculaceae, Bacteroides, Helicobacter* and *Akkermansia muciniphila*) in the fecal. Increased levels of acetate, propionate, and butyrate decrease the abundance of *Blautia, Lachnospiraceae, Rikenella,* and *Desulfovibrio* [153]. Cyanidin directly acts on the TLR4/NF-κB pathway to reduce the levels of typical proinflammatory markers (such as TNF-α, MCP-1, and IL-6) while also reducing the production of ROS and increasing the activity of various antioxidant enzymes, thereby promoting the metabolism and consumption of ROS. Additionally, cyanidin enhances insulin sensitivity by acting on the Akt and AMPK pathways, which is important for alleviating MetS [154].

Delphinidin, a purple plant pigment from *Consolida ajacis, Medicago sativa L.*, etc., exhibits a wide range of beneficial activities, including anticancer, anti-inflammatory, antihypertensive, antidiabetic, antiosteoporotic, antiviral, cardioprotective, and neuroprotective activities, as well as beneficial effects on nasal polyps and psoriasis [155]. Delphinidin significantly promotes the proliferation of *Bifidobacterium* spp., *Lactobacillus-Enterococcus, Akkermansia muciniphila, Bifidobacterium*, *Parabacteroides,* and *Lachnospiraceae* and inhibits the growth of the pathogens *Clostridium histolyticum, Erysipelotrichaceae,* and *Staphylococcus* [152, 155–157]. Delphinidin reversed HFD-induced metabolic endotoxemia; downregulated hepatic TLR2 and TLR4 expression; inhibited NF-κB, ERK1/2, AP-1, HIF-1, MCP-1 and TNF-α expression; protected pancreatic β cells from high-glucose-induced injury; decreased the production of superoxide; decreased the levels of iNOS and NADPH oxidases (such as NOX1 and NOX4); and modulated the AMPK/NOX/MAPK signaling pathway by increasing the phosphorylation of AMPKαto protect pancreatic β cells from high-glucose-induced injury [156].

Malvidin is sourced mainly from *Malva cathayensis M. G. Gilbert, Y. Tan*, and *Medicago sativa L.* Malvidin and its glycosides have found to have favorable in vitro antioxidant, anti-inflammatory, and angiotensin I-converting enzyme (ACE) inhibitory effects [196]. Malvidin inhibits the growth of harmful bacteria by lowering the pH value in the intestine and increasing the content of SCFAs, thereby increasing the abundance of *Enterococcus, Faecalibaculum, Lactobacillus, Prevotella, Bifidobacterium*, and *Ruminococcus* and reducing the relative abundance of *Escherichia Shigella* and *Streptococcus* in the intestinal tract [158]. Malvidin increases lysosomal function via transcription factor EB (TFEB), interacts with TFEB and activates the Nrf2/ARE and LAMP1 signaling pathways, effectively reversing FFA-induced lipid deposition in hepatocytes and increasing the activity of SOD, GPx, and CAT [159].

##### Biflavone

As shown in Fig. 3, ginkgetin is a natural and nontoxic flavonoid constituent that has been shown to exhibit anticancer, anti-inflammatory, antimicrobial, antiadipogenic and neuroprotective activities [197]. After intervention with *Ginkgo biloba* leaf extract, the abundance of probiotic bacteria, such as *Bifidobacterium, Lactobacillus,* and *Enterococcus*, in the intestinal tract increased [160]. Lian et al. [161] demonstrated that ginkgetin could attenuate lipid deposition in the aorta of rats with atherosclerosis, reduce the mRNA and protein expression of MMP-2 and MMP-9 in the thoracic aorta of rats, and exert an antiatherosclerotic effect by affecting the MMP and NO/NOS system. Ginkgetin inhibits STAT5 activity to block preadipocyte differentiation into adipocytes, thus inhibiting the expression of PPARγ and C/EBPα, which can effectively inhibit adipogenesis and alleviate MetS [162].

#### Modulation of the gut microbiota by nonflavonoid components

##### Polyphenols

As shown in Fig. 4, gallic acid (GA), also known as 3,4,5-trihydroxybenzoic acid, is a type of phenolic acid that is mainly derived from the TCMs *Galla chinensis, Corni fructus,* and *Rheum palmatum L.*. It is a natural polyphenol with the simplest chemical structure occurring in nature [198]. GA specifically protects the cardiovascular and nervous systems and exerts antidiabetic, antifibrotic, antitumor, antibacterial, antiviral, and anti-inflammatory effects [199]. As shown in Table 2, Li et al. [199] treated DSS-induced ulcerative colitis (UC) mice with GA, which altered the composition of intestinal microorganisms, as evidenced by an increase in the abundance of beneficial bacteria such as *Blautia, Ruminococcus, Lactobacillus* and *Prevotella* and a decrease in the abundance of pathogenic bacteria such as *Clostridium* and *Eubacterium*. Furthermore, in HFD-fed mice, GA treatment increased the expression of PPARγ in periepidymal white adipose tissue (EWAT), significantly enhanced Akt activation in EWAT, and contributed to the improvement of both insulin signaling and glucose tolerance [200].

**Table 2:** Effects of common phenolic acid components in traditional Chinese medicine polyphenols on the gut microbiota and metabolic syndrome

Representative component	Model	Dose	Duration	Changes in the gut microbiota	Mechanism	Reference
Gallic acid (GA)	adult male SD rats, DSS	6 mg/kg	8 days	*Blautia, Ruminococcus, Lactobacillus, Prevotella↑;Clostridium, Eubacterium↓*	↑PPARγ expression, ↑Akt activation, ↑insulin signaling, ↑glucose tolerance	[199, 200]
	5-week-old male C57BL/6 J mice, HFD	administered intraperitoneally 10 mg/kg/day	2 weeks			
Protocatechuic acid (PCA)	6-week-old male C57BL/6 J mice, HFD	HFD containing 0.4% (w/w) of PCA	12 weeks	*↓Roseburia, Lactobacillus, Enterorhabdus, Enterococcus, E. faecalis; ↑Akkermansia muciniphila, Rikenella, Turicibacter, Clostridium_sensu_stricto*,* Bifidobacterium*	↓TLR4/NF-κB, ↓CPT1α; ↑Fgf1, Igfbp2, Irs1, Irs2	[201, 202]
Chlorogenic acid (CGA)	8- to 9-week-old male Wistar rats, HFD	corn starch diet + chlorogenic acid (2 g/kg of food) and HFD + chlorogenic acid (2 g/kg)	8 weeks	*↓Lachnospiraceae, Ruminococcaceae, Streptococcus, Erysipelotrichaceae, Desulfovibrio; ↑Lactobacillaceae, Bacteroidaceae, Akkermansia muciniphila, Bifidobacterium, Streptococcus*	↑AMP, ↓TLR4, iNOS, COX-2 and NF-κB pathways; ↓mRNA expression of fatty acid synthase, acetyl-CoA, and stearoyl coenzyme A desaturase in the liver;↓ PPARγ, macrophage expression of TNF-α, MCP-1	[203, 204]
	8-week-old male C57BL/6 J mice, HFD	HFD + CGA (CGA, 150 mg/kg/BW)	14 weeks			
Caffeic acid (CFA)	3- to 4-week-old male C57BL/6 J mice, HFD	50 mg/kg BW	12 weeks	*↑Allobaculum, Bifidobacterium, Coriobacteriaceae_UCG-002, Lactobacillus, Mucinophilic Akkermansia muciniphila; ↓ Lachnospiraceae, Peptostreptococcaceae, Ruminococcaceae, Rhizobiales, Rhodobacterales, Sphingomonadales, Pseudomonadales, Arenimonas*	↓IL-1β, IL-6, TNF-α; ↑SOD1, GPX1, GPX2, CAT, IL-10; ↑Nrf-2, HO-1, NQO1	[205, 206]
Ferulic acid (FA)	HepG2 cells	50, 100, 200 μM	/	*↑Lachnospiraceae, Ruminococcus, Odoribacter, Prevotella, Bacteroides, Blautia, Faecalibacterium, Parabacteroides, Phascolarctobacterium, Akkermansia; ↓Alistipes, Desulfovibrio, Lactobacillus*	↑SCFAs, ↑ CPT-1, to enhance fatty acid oxidation, regulates HNF4α, p-FOXO-1, PPARγ, SREBP-1c, attenuates gluconeogenesis; ↓FAS-1, reduces adipogenesis	[207, 208]
	7-week-old male SD rats, streptozocin in citrate buffer intraperitoneally injected at 30 mg/kg BW on day 1, 3, 4	30 mg/kg BW/day	8 weeks			

*DSS* dextran sodium sulfate, *Fgf1* fibroblast growth factor 1, *Igfbp2* insulin-like growth factor-binding protein 2, *Irs* insulin receptor substrate, *COX-2* cyclooxygenase 2, *acetyl-COA* acetyl coenzyme A carboxylase, *NQO1* NAD(P)H quinone dehydrogenase 1, *HNF4α* hepatocyte nuclear factor 4 alpha, *FOXO-1* forkhead box O1, *SREBP-1c* sterol-regulatory element-binding protein 1c, *FAS-1* fatty acid synthase 1

Protocatechuic acid (PCA), formerly known as 3,4-dihydroxybenzoic acid, is a natural phenolic acid that is widely distributed in the daily diet and abundant in TCMs *Cibotii rhizoma* and *Carthami flos* [209]. In recent years, scientific studies have shown that PCA, a human metabolite of anthocyanins, exhibits a variety of pharmacological activities, including antioxidant, anti-inflammatory, antibacterial, antiviral, and anticancer properties, as well as protective effects on the central nervous system [210]. PCA and its parent anthocyanins also modulate the growth and abundance of specific gut microbiota. As shown in Table 2, Ajiboye et al. [201] reported that 600–800 mg/L PCA significantly increased superoxide dismutase, catalase and NAD/NADH, killing the conditionally pathogenic bacteria *Escherichia coli, Pseudomonas aeruginosa* and *Staphylococcus aureus*. In addition, PCA markedly reduced the abundance of *Roseburia, Lactobacillus, Enterorhabdus, Enterococcus,* and *E. faecalis* and increased the abundance of the beneficial bacteria *Akkermansia muciniphila, Rikenella, Turicibacter, Clostridium_sensu_stricto* and *Bifidobacterium*. As shown in Fig. 5, increasing the concentration of SCFAs in the intestine and body so that they are transported throughout the body via the bloodstream and bind to G protein-coupled receptors (GPRs) can reduce intestinal barrier inflammation and promote metabolism. PCA downregulates CPT1α and other genes related to insulin activity, downregulates the TLR4/NF-κB pathway, and increases lipocalin content, mitigating hepatic steatosis, by increasing the expression of Fgf1, Igfbp2, Irs1, and Irs2 [202].

Chlorogenic acid (CGA) is a condensed phenolic acid (5-O-caffeoylquinic acid) generated from caffeic acid and quinic acid, also known as coffee tannins, which naturally exist in TCMs, such as *Lonicerae japonicae flos* and *Eucommiae cortex* [211]. By affecting glucose metabolism, CGA exerts beneficial effects in obesity and diabetes; due to its phenolic hydroxyl structure, it reacts extremely easily with free radicals to exert antioxidant effects. CGA also exhibits antibacterial, antiviral, and antihypertensive effects [198, 212]. As shown in Table 2, CGA inhibits the growth of *Ruminococcaceae*, *Streptococcus, Erysipelotrichaceae* and *Desulfovibrio*; promotes the growth of *Lactobacillaceae, Bacteroidaceae*, *Akkermansia muciniphila*, *Bifidobacterium* and *Streptococcus*; activates AMP kinase; and decreases inflammatory cytokine expression and TLR4, iNOS, COX-2, and NF-κB pathway activity without affecting glucose tolerance or lipid concentrations [203]. CGA reduces hepatic lipogenic enzyme mRNA expression along with macrophage expression of TNF-α, MCP-1, and chemokine associated with inflammatory cascade pathways, thereby reducing fat deposition in adipose tissue, relieving TMAO-exacerbated atherosclerosis, and maintaining cardiac and hepatic structure and function [204].

Caffeic acid (CFA) is mainly found in the TCMs *Cimicifugae rhizoma* and *Eucommiae cortex*. 1,4-Benzodioxanes produced by self-oxidation of small-molecule phenolic acid constituents, such as caffeic acid, have anti-inflammatory, antitumor, adrenergic α-receptor antagonist, and antiviral activities [213]. As shown in Table 2, CFA improves the distribution of the microbiota during the growth process of DIO mice by increasing the abundance of probiotic bacteria such as *Allobaculum, Bifidobacterium, Coriobacteriaceae_UCG-002, Lactobacillus* and *Akkermansia muciniphila*, increasing energy expenditure, and inhibiting the reproduction of *Lachnospiraceae, Peptostreptococcaceae, Ruminococcaceae, Rhizobiales, Rhodobacterales, Sphingomonadales, Pseudomonadales,* and *Arenimonas* [205]. In female ICR mice, the mRNA expression of IL-1β, IL-6 and TNF-α was significantly downregulated, whereas the expression of SOD1, GPX1, GPX2, CAT, and IL-10 was upregulated after the administration of CFA. CFA supplementation significantly increased the mRNA expression of Nrf-2, HO-1, and NQO1, and it was hypothesized that it may activate the Nrf-2/HO-1 pathway to achieve antioxidant and anti-inflammatory effects. In addition, CFA supplementation can prevent intestinal barrier damage by increasing occludin gene expression [206].

Ferulic acid (FA) is widely found in the TCMs *Ferulae resina, Angelicae sinensis radix*, and *Chuanxiong rhizoma*. FA has a strong antioxidant effect and is capable of scavenging hydrogen peroxide, hydroxyl radicals, superoxide radicals, and peroxynitrite. FA has antimicrobial and anti-inflammatory properties, prevents coronary heart disease, exerts anticancer effects, protects the ovaries and inhibits liver damage [214]. After FA enters the digestive tract, it leads to an enrichment of bacterial genera such as *Lachnospiraceae, Ruminococcus, Odoribacter, Prevotella, Bacteroides, Blautia, Faecalibacterium, Parabacteroides, Phascolarctobacterium* and *Akkermansia* and a decrease in the abundance of *Alistipes, Desulfovibrio* and *Lactobacillus*. The mechanism of FA action in HepG2 cells involves the activation of the insulin/insulin-like growth factor-1 receptor-PI3K-Akt pathway, which subsequently regulates hepatic nuclear factor α (HNF4α), p-forkhead transcription factor-1 (p-FOXO-1) PPARγ, and sterol regulatory element-binding protein-1c (SREBP-1c), inactivates glucose-6-phosphatase, and further reduces phosphoenolpyruvate carboxykinase (PEPCK) expression. PEPCK further attenuates gluconeogenesis and reduces lipogenesis not only by lowering levels of fatty acid synthase-1 (FAS-1) and phosphorylating acetyl-CoA carboxylase (ACC) but also by increasing carnitine palmitoyltransferase (CPT) and palmitoyltransferase 1 (CPT-1) levels to increase fatty acid oxidation [207, 208].

##### Lignans

Schisandrin is derived from the lignans, the active ingredient of *Schisandrae chinensis fructus* and has multiple pharmacological effects, including anti-inflammatory, neuroprotective, and cell metabolism regulatory effects [215]. As shown in Table 3, schisandrin enters the gastrointestinal tract and causes a decrease in the abundance of *Ruminococcus* and an increase in the abundance of *Roseburia, Prevotella, Bacteroides, Lachnospiraceae, Lactobacillus, Alloprevotella, Bifidobacterium* and *Akkermansia muciniphila* [216]*.* Fan et al. [217] reported that schisandrin activates the AMPK/mTOR pathway to induce autophagy and reduces the quantity of lipid droplets in FFA-treated HepG2 cells and MPHs. Schisandrin induces the expression of pregnane X receptor (PXR) target genes in the liver and activates hPXR genes such as CYP3A4, UGT1A1 and organic anion transporter polypeptide 2 (OATP2), not only significantly preventing bile acid-induced intrahepatic cholestasis and hepatic necrosis but also markedly reducing the levels of ALT, AST, ALP, serum total bile acids (TBA), and total bilirubin, accelerating the metabolism of bile acids [218].

**Table 3:** Effects of lignin, stilbenoids and other common components of traditional Chinese medicine polyphenols on the gut microbiota and metabolic syndrome

Representative component	Model	Dose	Duration	Changes in the gut microbiota	Mechanism	Reference
Schisandrin	male SD rats	280 mg/kg	5 weeks	*↓Ruminococcus, ↑Roseburia, Prevotella, Bacteroides, Lachnospiraceae, Lactobacillus, Alloprevotella, Bifidobacterium, Akkermansia muciniphila*	↑ AMPK/mTOR pathway, PXR, hPXR genes (CYP3A4, UGT1A1 and organic anion transporter polypeptide 2 (OATP2)); ↓TBA, Tbil	[216–218]
	8–10-week-old C57BL/6 J mice	100 mg/kg	7 days			
	FFA-stimulated HepG2 cells and MPHs	/	/			
	male C57BL/6 J mice, HFD	50 mg/kg/d BW	5 weeks			
Resveratrol (RSV)	6-week-old male C57BL/6 J mice, HFD	300 mg/kg/day trans-RSV	16 weeks	*↑Blautia, Alloprevotella, Lactobacillus, Bifidobacterium; ↓Lactococcus lactis, Clostridium, Oscillibacter, Hydrogenoanaerobacterium, Enterococcus faecalis, Desulfovibrio*, *Lachnospiraceae_NK4A136_group, Ruminiclostridium_9*	↑AMPK, SIRT1, PGC-1α; activates AMPK and Nrf2	[219–222]
	28 adults aged 68 ± 7 years (84% male) with stage 3 CKD and DM	400 mg/d	6 weeks			
	19 women and 18 men with normal glucose tolerance and BMI > 25 kg/m^2^	EGCG + RES, 282 + 80 mg/d	12 weeks			
Pterostilbene	Zucker (fa/fa) rats;	diet supplemented with 15 mg/kg BW/day	6 weeks	*↑Verrucomicrobia, Akkermansia, Bacteroides*, *Odoribacter, O. splanchnicus, A. shahii; ↓Firmicutes, Lachnospiraceae, Phascolarctobacterium, Negativicutes*	↑SIRT1/AMPK, ↑FA β oxidation, AMPK, CPT-1, CPT-2, PPARα; ↓PPARγ, SREBP-1c	[223–225]
	BMI 25.0–39.9 kg/m^2^ and HFF ≥ 15% by MRIPDFF	4 NRPT capsules	26 weeks			
Curcumin	8-week-old male C57BL/6 J mice, HFD	100 mg/kg/d, dissolved in 0.5% carboxymethylcellulose, intragastric gavage	4 weeks	*↓Rikenella, Prevotella, Coriobacterales, Ruminococcus; ↑Prevotella, Bacteroides*	↑Glut4, Akt phosphorylation levels in peripheral tissues; ↓Pck1, G6pc, NF-κB p65	[226–229]
	adult male albino Wistar rats, HFD or 35 mg/kg BW STZ	35 mg/kg BW	8 weeks			
	Diagnosed NASH patients 18–70 years of age	phospholipid curcuminoids 2 g/d	72 weeks			
Psoralene (PSO)	LO2, IEC-6, 293 T cells	250 μg/mL, 375 μg/mL, 500 μg/mL	/	*↑Akkermansia muciniphila, Norank_F_Muribaculaceae; ↓Escherichia-Shigella*	↓IL-6, IL-1, TNF-α, PPARγ	[230–232]
	New Zealand white rabbits	45 mg/kg	3 days			
	male C57BL/6 SPF mice, 2.5% DSS	10 mg per kg body weight, at a volume of 300 µL	30 days			

*FFA* free fatty acid, *MPHs* mouse primary hepatocytes, *mTOR* mammalian target of rapamycin, *PXR* pregnane X receptor, *CYP3A4* cytochrome P450 family 3 subfamily A member 4, *UGT1A1* UDP glucuronosyltransferase family 1 member A1, *OATP2* organic anion transporter polypeptide 2, *TBA* total bile acid, *Tbil* total bilirubin, *CKD* chronic kidney disease, *DM* diabetes mellitus, *SIRT1* silent information regulator 1, *PGC-1α* peroxisome proliferation-activated receptor γ coactivator-1α, *MRIPDFF* magnetic resonance imaging-proton density fat fraction, *FA β oxidation* fatty acid beta oxidation, *NASH* nonalcoholic steatohepatitis, *Pck1* phosphoenolpyruvate carboxykinase 1, *G6pc* glucose-6-phosphatase G-6-pase, *LO2* human normal liver cells, *IEC-6* rat small intestine crypt epithelial cells, *293 T* human embryonic kidney cells

##### Stilbenoids

As shown in Fig. 4, resveratrol (RSV) is a polyphenol compound with a diphenylethylene structure, mostly from the TCMs *Cassiae semen* and *Polygoni cuspidati rhizoma*. Resveratrol exhibits antioxidant, cardioprotective, antifatigue, antiaging, anticancer, antimicrobial, anti-inflammatory, and antiviral effects and ameliorates the pharmacological effects of Alzheimer’s disease [22, 233]. As shown in Table 3, a group of controlled clinical trials revealed that in 28 adults (68 ± 7 years old, 84% male) with stage 3 CKD and diabetes, 400 mg/day RSV supplementation for 6 weeks increased blood flow-mediated vasodilation and improved endothelial function [22]. In addition, 19 women and 18 men with normal glucose tolerance and BMI > 25 kg/m^2^ participated in a randomized, double-blind trial. After 12 weeks of treatment with EGCG + RES (282 + 80 mg/day), the diet-induced improvement in MetS associated with the gut microbiota was more easily observed in obese or overweight men, confirming sex differences in cardiovascular metabolic improvement [219]. In HFD-fed mice, the abundance of *Blautia* and *Alloprevotella*, which produce SCFAs, was increased by RSV supplementation, which restored HFD-induced decrease in the abundance of the mouse intestinal species *Desulfovibrio*, *Lachnospiraceae_NK4A136_*group and *Ruminiclostridium_9*. In addition, RSV can suppress obesity-related gut microbes, such as *Lactococcus lactis, Clostridium, Oscillibacter* and *Hydrogenoanaerobacterium*, significantly increasing the abundance of *Lactobacillus* and *Bifidobacterium*, which are negatively correlated with weight, and decreasing the abundance of *Enterococcus faecalis*, which is positively associated with body weight [220]. RSV may serve as a cardioprotective, antioxidant and anti-inflammatory agent by targeting several major metabolic sensor/effector proteins (e.g., AMPK, SIRT1, and peroxisome proliferator-activated receptor γ coactivator-1α (PGC-1α)) that regulate NF-κB in inflamed adipose tissue [221, 222].

Pterostilbene (PT) is a methylated derivative of resveratrol and exists as a trans-resveratrol analog. Pterostilbene is primarily found in the TCM *Pterocarpus indicus Willd.* and was initially isolated from *Santali albi lignum*. Modern pharmacological studies have shown that pterostilbene exerts antitumor, anti-inflammatory, antioxidant, antiaging, and cardiovascular protective effects similar to those of resveratrol [234, 235]. As shown in Table 3, a 6-month controlled clinical trial to evaluate the efficacy of daily supplementation with NRPT (a combination of nicotinamide riboside and pterostilbene containing 125 mg of NR and 25 mg of PT per capsule) in 111 adult patients with MASLD demonstrated that it reduces hepatic inflammatory markers of MASLD at normal dosages and can effectively alleviate MASLD [223]. Etxeberria et al. [224] suggested that PT causes changes in the composition of the gut microbiota, thereby increasing insulin sensitivity. Reduced abundance of *Firmicutes, Phascolarctobacterium, Negativicutes*, and *Lachnospiraceae* and increased levels of *Verrucomicrobia, O. splanchnicus*, *A. shahii, Akkermansia*, *Bacteroides* and *Odoribacter* was detected in the PT group, confirming that pterostilbene-induced amelioration of obesity in rats was associated with enrichment of *Akkermansia* and *Odoribacter*. Pterostilbene supplementation protects TJ proteins through the NF-κB-mediated MLCK-pMLC pathway; maintains the intestinal epithelial barrier; markedly activates the SIRT1/AMPK signaling pathway; enhances FA β oxidation; increases AMPK activation and the expression of CPT-1, CPT-2, and PPARα; and further downregulates the expression of PPARγ and SREBP-1c in the livers of HFD-fed mice, resulting in improved glucose homeostasis [225].

##### Others

As shown in Fig. 4, curcumin is a diketone phenolic yellow pigment derived from *Zingiberaceae Curcuma aromatica Salisb., C. longa L., C. zedoaria (Berg.) Rosc., Araceae Juss. Acorus calamus L.,* and *Araceae Juss* [236]. Its pharmacological effects include anti-inflammatory, choleretic, antibacterial, antitumor, hypolipidemic, antioxidant and immunomodulatory effects [125]. As shown in Table 3, 52 patients with biopsy-proven NASH were randomized at a 1:1 ratio to receive 2 g/d phospholipid curcuminoids for 72 weeks. The treatment was well tolerated and highly efficacious, possibly improving hepatic histology and suppressing NASH through the inhibition of NF-κB, mitigating renal disease, and improving metabolic disturbances [226]. Zhong et al. [227] concluded that curcumin might affect fibroblast growth factor 15 (FGF15) expression by regulating the gut microbiota, thereby increasing insulin sensitivity and alleviating IR in HFD-fed mice. A previous study revealed that curcumin markedly increased Akt phosphorylation levels in peripheral tissues by decreasing *Rikenella* abundance; increasing *Prevotella* and *Bacteroides* abundance; decreasing the mRNA expression levels of Pck1 and G6pc, which encode two key enzymes involved in hepatic gluconeogenesis; and inhibiting lipopolysaccharide-induced NF-κB p65 translocation and mitogen-activated protein kinase phosphorylation in dendritic cells, thereby reducing inflammation [125, 228]. In addition, curcumin reduces the abundance of microbial species associated with cancer, such as *Prevotella, Coriobacterales,* and *Ruminococcus* [229].

Psoralene (PSO) is a furanocoumarin analog in the TCM *Ficus pandurata Hance*. The pharmacological effects of PSO include antitumor, anti-HIV, antioxidant, antimicrobial, anti-inflammatory, analgesic, antidepressant, and anticancer effects [237–240]. As shown in Table 3, Zhang et al. [230] reported that PSO can reduce the abundance of *Escherichia-Shigella* by increasing the abundance of the dominant bacterial genera *Akkermansia muciniphila* and *Norank_F_Muribaculaceae*, as well as by promoting intestinal bile acid metabolism and increasing the expression of certain bile acid transporters, thereby lowering the levels of inflammatory factors (IL-6, IL-1β,and TNF-α) and affecting the integrity of the intestinal barrier. In addition, PSO can reduce the protein expression level of PPARγ, increase osteocalcin expression, reduce lipid synthesis in bone marrow cells, and improve steroid-induced ischemic necrosis of the femoral head [231, 232].

## Conclusion and prospects

At present, the treatment for MetS mainly comprises comprehensive approaches, including lifestyle changes, weight loss, and management of blood sugar, blood pressure, blood lipids, and clotting risk, among others [241, 242]. At the same time, clinicians often adopt comprehensive multidrug treatments. However, there are still problems such as poor patient compliance, difficulty in taking medication, long medication cycles, and high costs. Owing to the complexity of the mechanisms underlying MetS, it is often difficult to achieve satisfactory results with single-target interventions. TCM has a history of 5000 years, has the advantages of multiple components, multiple targets and multiple signaling pathways, and involves the same pathways and targets as Western drugs do, and these advantages may compensate for the shortcomings of conventional MetS treatments [243]. Moreover, most TCM polyphenols are derived from plants, which are safer than traditional medicines, have a variety of routes of administration, and improve MetS comprehensively. However, TCM involves different treatments for the same disease and the same treatment for different diseases; thus, the clinical application of TCM still needs much experience.

The gut microbiota, as a factor affecting human health, can regulate the intestinal microecological balance, maintain the stability of the intestinal barrier, influence the maintenance of host metabolic functions, maintain the development of the immune system and resist the invasion of intestinal pathogens. Intestinal microecological dysregulation is closely related to the occurrence of MetS. Available evidence suggests that polyphenolic compounds in TCMs regulate the intestinal microecology by interacting with the gut microbiota. On the one hand, TCM polyphenolic compounds regulate intestinal metabolites and influence bacterial growth, gut microbiota abundance and intestinal barrier stabilization. On the other hand, the gut microbiota can decompose TCM polyphenols to produce metabolites, which in turn regulate the microbial community. The available data suggest that the regulatory effect of TCM polyphenols on gut microbiota stabilization may play an important role in the treatment of MetS.

TCM polyphenols improve MetS mainly through the following pathways. I. TCM polyphenols increase antioxidant levels, inhibit lipid accumulation, and suppress inflammation [244]. II. TCM polyphenols activate AMPK, which can increase glucose uptake by promoting the movement of GLUT1 and GLUT4 from the intracellular membrane to the cytoplasmic membrane; additionally, AMPK can activate the SREBP-1C, Nrf2, and PPAR pathways and competitively inhibit the NF-κB, AP-1, and JAK-STAT pathways in the inflammatory response, thus increasing antioxidant levels and inhibiting lipid accumulation, thus suppressing inflammation. Second, TCM polyphenols activate the IR-related and PI3K/Akt signaling pathways, affecting glucose and lipid metabolism and maintaining blood glucose stability [245]. TCM polyphenols inhibit the TLR pathway, inhibit IκB kinase β (IKKβ), and inhibit NF-κB, alleviating inflammatory responses and improving abnormalities in lipid metabolism and insulin activity [246]. III. TCM polyphenols can promote the production of SCFAs by intestinal SCFA-producing bacteria, bind to GPRs, participate in the generation and repair of intestinal mucosal cuprocytes, inhibit the MAPK/NF-κB/STAT3 pathway, reduce the production and release of inflammatory factors, and alleviate intestinal barrier inflammation [247]. The regulation of gut microbiota stabilization by TCM polyphenols is important in the treatment of MetS and may be used as a new approach for the treatment of MetS.

This paper summarizes the effects of gut microbiota metabolites on MetS on the basis of the literature and discusses the mechanisms by which TCM polyphenols affect MetS, confirming that TCM polyphenols exert certain therapeutic effects against MetS that are related to the modulation of the structure and function of the gut microbiota. However, TCMs often contain hundreds of ingredients, and the polyphenols in TCMs are diverse and complex in structure. Thus, TCMs produce complex metabolites after entering the human body and reacting with the gut microbiota, and further experimental verification is needed to explore the role of TCM polyphenols in the regulation of the gut microbiota and the mechanisms influencing the development of MetS. Research on the therapeutic mechanisms of TCM polyphenols in the treatment of MetS is still insufficient, and there are limitations in the current study, such as the existence of thousands of TCM prescriptions for diseases, the phenomenon of different treatments existing for the same disease and the same treatments being used for different diseases, and the variability of TCM formulas, dosages, and individual responses, which are currently still bottlenecks in the use of TCM polyphenols in the treatment of MetS. In future studies, we should explore the synergistic effects of different TCM polyphenols, the combination of TCM polyphenols with existing MetS treatments, and the influence of lifestyle factors on the efficacy of herbal medicines.

## Data Availability

Not applicable.
